# An African swine fever virus-specific antibody reactome reveals antigens as potential candidates for vaccine development

**DOI:** 10.1128/jvi.00478-25

**Published:** 2025-08-14

**Authors:** Songxin Guo, Yi Ru, Hui Zhang, Junbiao Xue, Huanan Liu, Dong Men, Zongqiang Cui, Chaochao Shen, Hong Tian, Chun Ma, Jun Gong, Jintian Xu, Dianbing Wang, Rui Gong, Xiaowei Zhang, Heng Rong, Yan-Yi Wang, Chenli Liu, Zhuojun Dai, Shengce Tao, Jiaoyu Deng, Haixue Zheng, Feng Li, Xian-En Zhang

**Affiliations:** 1 Key Laboratory of Virology and Biosafety, Wuhan Institute of Virology, Chinese Academy of Sciences 74614 , Wuhan, China; 2 University of Chinese Academy of Sciences 74519, Beijing, China; 3 State Key Laboratory for Animal Disease Control and Prevention, College of Veterinary Medicine, Lanzhou University, Lanzhou Veterinary Research Institute, Chinese Academy of Agricultural Sciences 111658 , Lanzhou, China; 4 Key Laboratory of Systems Biomedicine (Ministry of Education), Shanghai Center for Systems Biomedicine, Shanghai Jiao Tong University 12474, Shanghai, China; 5 Guangzhou Laboratory 612039, Guangzhou, China; 6 National Laboratory of Biomacromolecules, Institute of Biophysics, Chinese Academy of Sciences 578670 , Beijing, China; 7 Institute of Synthetic Biology, Shenzhen Institute of Advanced Technology, Chinese Academy of Sciences 85411, Shenzhen, China; 8 Faculty of Synthetic Biology, Shenzhen University of Advanced Technology 704566, Shenzhen, China; Lerner Research Institute, Cleveland Clinic, Cleveland, Ohio, USA

**Keywords:** African swine fever virus, proteome microarray, humoral immunity, live-attenuated vaccines, B-cell antigens

## Abstract

Developing an efficient and safe vaccine for African swine fever (ASF), a devastating disease of pigs, remains a significant challenge mainly due to limited knowledge of the immune correlates of protection. Identifying protective determinants is difficult because ASF virus (ASFV) is a large and complex DNA virus encoding over 160 proteins. Here, we constructed an ASFV proteome microarray containing 160 full-length proteins for profiling ASFV-specific antibodies. An antibody reactome containing 46 ASFV proteins (including 12 newly recognized B-cell antigens) was established by analyzing several cohorts of serum samples from pigs protected with different live-attenuated vaccines (LAVs). A proteome-wide study of antibody dynamics over a 26-day period provided a multi-dimensional landscape of the host humoral response against ASFV after acute infection, LAV immunization, and post-vaccination challenge. This study provides a comprehensive understanding of ASFV-induced humoral immune responses, highlights B-cell antigen candidates for vaccine design, supports the investigation of LAV protection mechanisms, and would accelerate vaccine development.

**IMPORTANCE:**

African swine fever (ASF) poses a severe threat to global swine industries, with vaccine development hindered by limited understanding of immune protection. A comprehensive understanding of antibody responses against ASF virus (ASFV) and the discovery of protective antigens are fundamental to vaccine development. This study constructed an ASFV proteome microarray to profile antibody responses against 160 viral proteins and established the antibody spectra against ASFV with dynamic features. The proteome microarray offers a high-throughput platform for understanding ASFV immunology and pathogenicity and will contribute to ASF vaccine development and diagnosis.

## INTRODUCTION

African swine fever (ASF) is an acute, hemorrhagic, and highly contagious infectious disease with a mortality rate of up to 100% (1). Since the first report of ASF in 1921 in Africa, it has spread across continents to more than 50 countries and regions. ASF was transmitted into China in August 2018, and by 4 November of the same year, it had already caused the death of more than 100,000 pigs, leading to an estimated loss exceeding $20 million (2). From 2018 to 2019, more than 30 million pigs were culled, resulting in an estimated economic loss of $2 billion in the global farming industry (3).

African swine fever virus (ASFV), the causative agent of devastating ASF, is the only member of the genus *Asfivirus* within the family *Asfarviridae* (4). It has a linear double-stranded DNA genome of 170–193 kb that contains 160–175 open reading frames (5, 6). The virion possesses a complex and multilayered structure composed of at least 68 virus-encoded proteins (3, 7–9). Vaccination is a prioritized means to control the epidemic of infectious diseases. Numerous efforts have been devoted to ASFV vaccine development. However, there are still no safe and effective ASFV subunit or vectored vaccines available. Although live-attenuated vaccines (LAVs) have shown potential with complete protection, such as those commercialized in Vietnam (10), safety concerns exist (11). Subunit vaccines combining multiple antigens are believed to be a more promising strategy (12, 13).

Increasing evidence shows that humoral immunity plays a critical role in effectively preventing ASFV invasion (4, 14–17). Many ASFV proteins have been found to induce a humoral immune response and have been tested for vaccine development. For example, B646L encoding p72 protein, EP402R encoding CD2v protein, CP204L encoding p30 protein, and E183L encoding p54 protein can induce neutralizing antibodies against genotype I ASFV strains (18–20). However, vaccines developed based on these known immunogens cannot provide satisfactory protection, and the protective effect varies across different genotypic and serotypic strains (13, 21–24). A combination of multiple immunogens is required for a high-efficacy ASFV vaccine (13). However, there is a lack of comprehensive understanding of humoral immunity against ASFV in infected pigs, let alone establishing a combination of multiple immunogens that can offer satisfactory protection. Being a large DNA virus, the large number of viral proteins and the complexity of ASFV virions make it difficult to profile the specific antibody spectrum in infected or LAV-protected pigs, which lays the foundation for identifying protective antigens (25).

Protein microarrays are systematic protein analysis tools characterized by high sensitivity, miniaturization, and high-throughput processing (26, 27). In principle, they can be used for identifying drug targets, antigens, and protein-protein interactions, all of which are high throughput. In the analysis of serum antibodies after pathogen infection, protein microarrays can be used to determine the specific serum antibody profile, analyze the dynamic changes in serum antibodies, and identify new antigens (28–31). The serum antibody spectrum built with protein microarrays further provides insights into the relationship between immune protection and antibody responses (28, 30, 32, 33). This knowledge is instrumental in addressing the challenges of vaccine design and diagnostics for complex pathogens such as ASFV.

In this study, we built a protein expression library of ASFV in *Saccharomyces cerevisiae* after several years of effort and then constructed an ASFV whole proteome microarray containing 160 purified ASFV proteins. Using this high-throughput analysis tool, we profiled the longitudinal antibody spectrum in pigs infected with a genotype II ASFV isolate and two attenuated mutants. The main results include the following: (i) the first antibody spectrum against ASFV is established; (ii) 46 antigens are shown to be recognized by sera from pigs completely protected by LAVs, 12 of which are found serum reactive for the first time; and (iii) the temporal dynamics of antibody levels is depicted, revealing varied immune response patterns of different antigens and highly coincident immune responses of certain ASFV proteins.

## RESULTS

### Construction and characterization of the ASFV proteome microarray with 160 proteins

To construct the ASFV protein library for microarray analysis, we cloned 168 open reading frames (ORFs) of genotype II ASFV-SY18, which was the first strain isolated in China in 2018 and subsequently became prevalent. To obtain soluble ASFV proteins, we used the *Saccharomyces cerevisiae* expression system (34). The purification protocol took advantage of both a 96-well format and immobilized affinity chromatography. Some proteins exhibited high expression levels, allowing for one-step purification, while others with lower expression levels necessitated expanding the yeast culture volume and post-purification concentration. Finally, we obtained an ASFV protein library with 95% proteome coverage containing 160 ASFV-encoded proteins (Fig. S1), with 8 proteins (MGF_110_11L, MGF_100_1L, MGF_110_9L, MGF_505_4R, MGF_505-1R, MGF_505-11L, I329L, and NP1450L) not included due to failure of expression.

**Fig 1:**
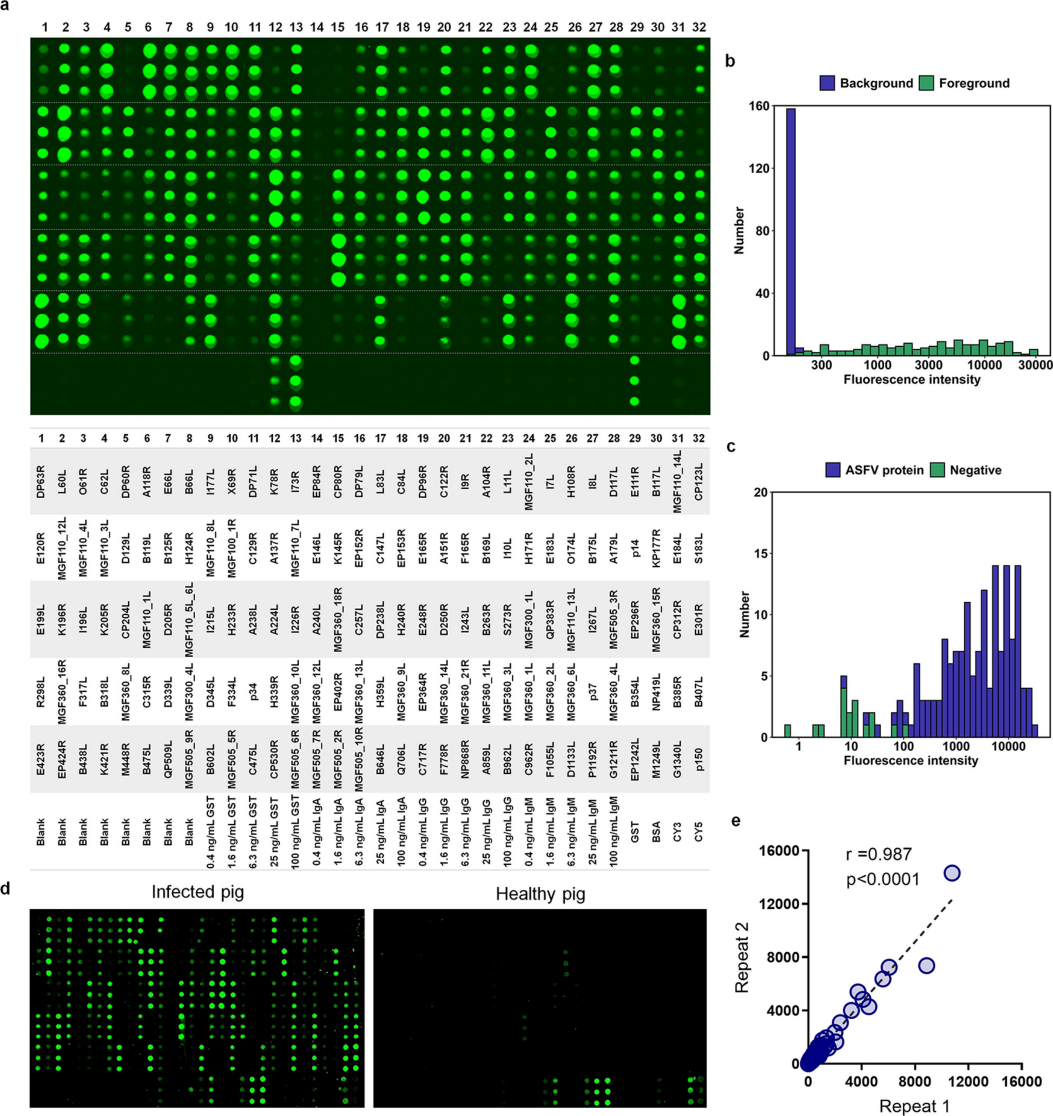
Construction and characterization of the ASFV proteome microarray. In parallel with the controls, a total of 160 GST-tagged ASFV proteins were printed in triplicate onto a PATH substrate slide, generating 14 identical subarrays per slide. The slides were probed with an anti-GST antibody and a Cy3-labeled secondary antibody. (a) A representative subarray (upper) and the layout of the array (lower). (b) Histogram analysis of the fluorescence intensity of all the immobilized ASFV proteins based on their N-terminal GST tag probed with an anti-GST antibody and a fluorescently labeled secondary antibody. (c) Fluorescence intensity distribution of the spots of ASFV proteins and negative controls (including blank, BSA, IgG, IgM, and IgA) after probing with an anti-GST antibody and a fluorescently labeled secondary antibody. (d) Representative subarrays probed with sera from an ASFV-infected pig and a healthy pig. The IgG signals are shown in green. (e) Correlation analysis showing the repeated experimental results for the same serum sample.

We then spotted the full set of purified recombinant proteins in triplicate on the microarray slide, accompanied by a set of controls, including elution buffer, bovine serum albumin (BSA), and three serially diluted subtypes of immunoglobulin control from pigs and glutathione S-transferase (GST) protein. Given that we tagged all the proteins with GST, we analyzed the entire set of immobilized proteins on the microarray by employing an anti-GST antibody for probing (Fig. 1a), one of the steps of the microarray qualification. The distributions of the foreground and background signal intensities of the 160 ASFV proteins were almost completely separate (Fig. 1b), and the signal intensity distributions for the ASFV proteins and negative controls were separated to a great extent (Fig. 1c), indicating that the vast majority of the printed spots on the microarray contained substantial amounts of recombinant proteins.

When examined with sera from infected pigs, the microarray showed multipoint antibody responses with varying signal strengths. In contrast, the sera from healthy pigs did not produce obvious signals in the microarray assay (Fig. 1d and Fig. S2). We also randomly selected one serum sample from an infected pig and probed it on two microarrays. The resulting high Spearman correlation coefficient (0.987) demonstrated excellent reproducibility in antibody profiling using our microarray (Fig. 1e). For biosafety reasons, we treated pig serum samples with heat inactivation. Notably, this process did not adversely affect the serum analysis (Fig. S3). Consequently, we successfully developed a high-performance ASFV proteome microarray for antibody profiling.

### Humoral immunoproteomes of a virulent ASFV strain and its attenuated mutants

Although LAVs are faced with safety concerns, the fact that LAVs can confer protection (22) supports that the protective B-cell antigens, if any, are among the antibody-positive viral proteins found by profiling the sera from LAV-immunized pigs. Previously, we tried to construct LAVs based on the highly virulent ASFV CN/GS/2018 strain by deleting different genes, resulting in varying effects on attenuation and immune protection. We finally achieved two LAV mutants with immunosuppressive gene deletions, which provide 100% homologous protection. One is ASFV-GS-Δ18R/NL/UK with deletions of three genes, MGF_360_18R, DP71L, and DP96R (35). The other is ASFV-GS-ΔMGF110/360-9L with deletions of two genes, MGF_110_9L and MGF_360_9L (36). To prepare serum samples for profiling the antibody spectrum, we infected pigs with the two LAV mutants and the virulent ASFV CN/GS/2018 strain. The two LAV infection groups allowed for comparative analyses and an extensive description of the elicited antibody responses.

**Fig 2:**
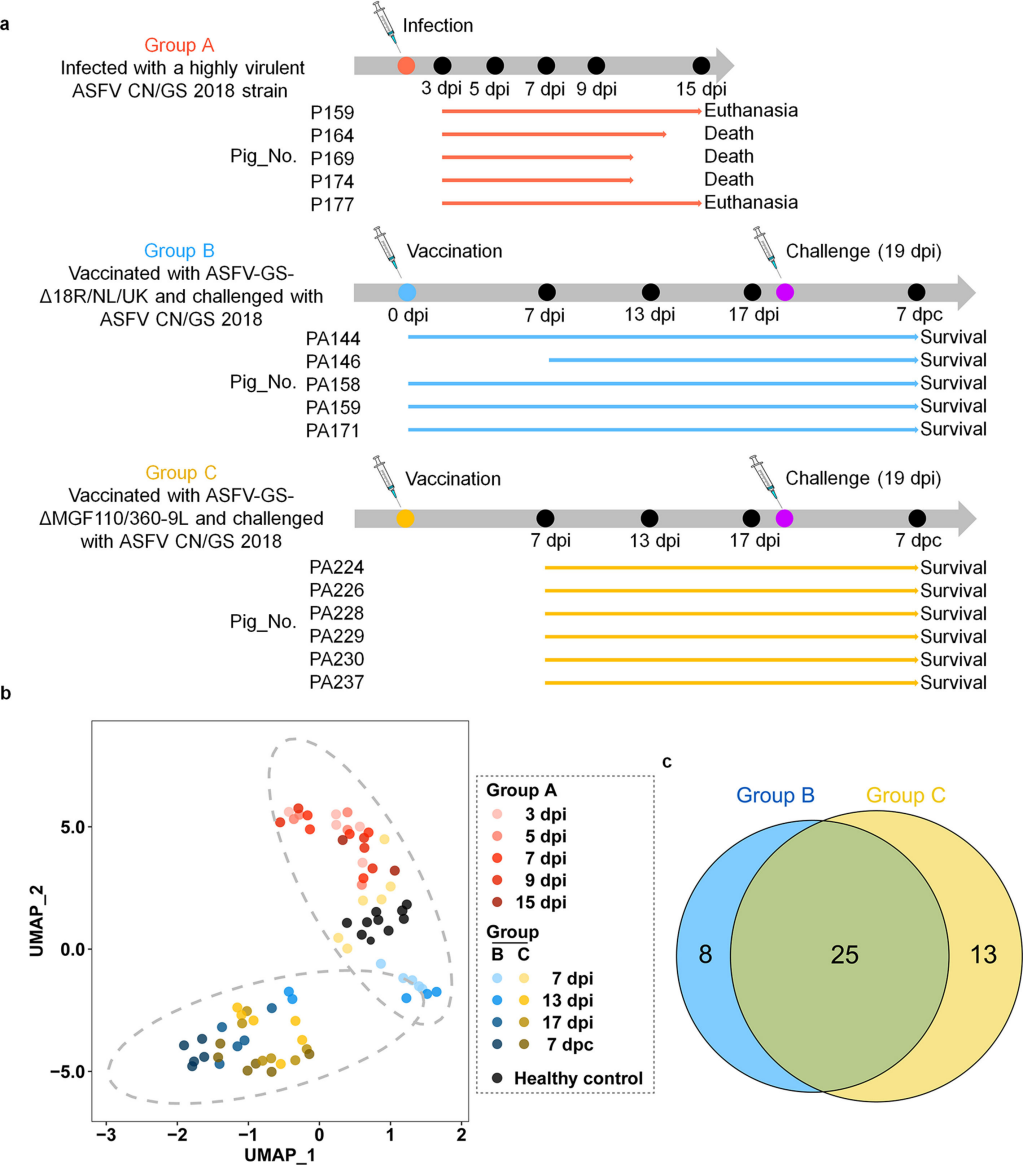
High-throughput analysis of sera from ASFV-infected pigs using a proteome microarray. (a) Timelines of animal treatment and sample collection. Group A pigs (*n* = 5) were infected with a virulent ASFV CN/GS 2018 strain at 1 HAD_50_. Three pigs died by 15 dpi, while the other two pigs developed clinical symptoms of ASF during the observation period but ultimately survived. Serum samples were collected at 3, 5, 7, 9, and 15 dpi. Group B pigs (*n* = 5) inoculated with 10^4^ HAD_50_ of ASFV-GS-ΔMGF360-18R/DP71L/DP96R survived the 17-day observation period and remained alive after being challenged with 10^2^ HAD_50_ of the parental virus during the 13-day observation period. Serum samples were collected at 0, 7, 13, and 17 dpi and at 7 dpc. Group C pigs inoculated with 10^4^ HAD_50_ of ASFV-GS-ΔMGF110/360-9L (*n* = 6) survived the 17-day observation period and remained alive after being challenged with 10^2^ HAD_50_ of the parental virus during the 13-day observation period. Serum samples were collected at 7, 13, and 17 dpi and 7 dpc. (b) Uniform manifold approximation and projection (UMAP) of the 160-ASFV-protein-specific IgG signals in the serum samples from the three groups. Each point represents an individual serum sample. (c) The amounts of the IgG-positive ASFV proteins and the shared portion from groups B and C.

As shown in Fig. 2a, the pigs were divided into three groups, A, B, and C. Group A (five pigs) was intramuscularly infected with one median hemadsorption unit (HAD_50_) of the highly virulent ASFV CN/GS/2018 strain. The pigs gradually developed symptoms related to ASF, and three of them died within 13 days. Serum samples were collected at 3, 5, 7, 9, and 15 days post-infection (dpi). Groups B (five pigs) and C (six pigs) were infected (immunized) with ASFV-GS-Δ18R/NL/UK and ASFV-GS-ΔMGF110/360-9L, respectively. During the 17-day observation period after immunization, pigs in groups B and C showed only temporary, slight increases in body temperature and no other obvious symptoms of ASF. At 19 dpi, the pigs were challenged by injecting a lethal dose of the parent virulent strain to check for protection. As in our previous reports (35, 36), all the pigs survived. Serum samples were collected before and after the challenge.

To comprehensively elucidate the characteristics of humoral immune responses in pigs after viral infection, we analyzed the collected serum samples using microarrays. The serum probing results of IgG signals for 160 ASFV proteins were processed with the uniform manifold approximation and projection algorithm for dimensionality reduction to provide an overview (Fig. 2b). The samples from group A at all time points and the two sets of groups B and C at 7 dpi clustered together with the healthy control group, indicating that group A exhibited consistently low or no antibody response levels throughout the experiment phase, while groups B and C showed weak antibody responses at the early stage of immunization. The samples collected at 13 dpi and later from groups B and C formed a distinct cluster, suggesting that the IgG antibody spectrum had changed due to immunization with the LAVs, and the changes were homogeneous between groups B and C.

To establish the humoral immunoproteome of ASFV, we prioritized the proteins that induced relatively high levels of antibodies by applying the analytical criterion described in the methods section. Group A pigs, infected with the virulent strain, did not exhibit any positive IgG response. This result is consistent with previous reports that pigs cannot develop IgG responses before death in acute ASFV infections based on detection using only a few selected ASFV proteins (37–39). Our microarray-based proteome profiling confirms that IgG responses are systematically deficient in acute ASFV infections. In contrast, pigs in groups B and C, which were infected with the attenuated mutants, showed substantial antibody responses to various viral proteins. Specifically, 33 and 38 IgG-positive proteins were identified in groups B and C, respectively. Thus, 46 IgG-positive proteins were detected in the two groups (Table 1). The positive antibody profiles in groups B and C were similar and shared a set of 25 positive proteins (Fig. 2c), which accounted for 75.8% and 65.8% of the positive proteins of groups B and C, respectively. As for IgM-positive viral proteins, no positive result was detected in group A, while a total of 21 IgM-positive proteins were identified in groups B and C (Table S1).

**TABLE 1:** IgG-positive ASFV proteins identified in groups B and C^*a*^

	Protein	Group B	Group C
1	**p150**	13 dpi, 17 dpi, and 7 dpc	7 dpi, 13 dpi, and 7 dpc
2	**p34**	7 dpi, 13 dpi, 17 dpi, and 7 dpc	7 dpi, 13 dpi, and 7 dpc
3	**E183L (p54)**	7 dpi, 13 dpi, 17 dpi, and 7 dpc	7 dpi, 13 dpi, 17 dpi, and 7 dpc
4	**B602L**	7 dpi, 13 dpi, 17 dpi, and 7 dpc	7 dpi, 13 dpi, 17 dpi, and 7 dpc
5	**CP204L (p30)**	7 dpi, 13 dpi, 17 dpi, and 7 dpc	7 dpi, 13 dpi, 17 dpi, and 7 dpc
6	**CP312R**	7 dpi, 13 dpi, 17 dpi, and 7 dpc	13 dpi, 17 dpi, and 7 dpc
7	**CP530R (pp62)**	7 dpi, 13 dpi, 17 dpi, and 7 dpc	13 dpi, 17 dpi, and 7 dpc
8	**D205R**	7 dpi, 13 dpi, 17 dpi, and 7 dpc	7 dpi, 13 dpi, 17 dpi, and 7 dpc
9	**H240R**	7 dpi, 13 dpi, 17 dpi, and 7 dpc	7 dpi, 13 dpi, 17 dpi, and 7 dpc
10	**KP177R (p22)**	7 dpi, 13 dpi, 17 dpi, and 7 dpc	13 dpi, 17 dpi, and 7 dpc
11	**B646L (p72)**	7 dpi, 13 dpi, 17 dpi, and 7 dpc	7 dpi, 13 dpi, 17 dpi, and 7 dpc
12	**MGF_110_5L_6L**	7 dpi, 13 dpi, 17 dpi, and 7 dpc	7 dpi, 13 dpi, 17 dpi, and 7 dpc
13	**EP402R (CD2v)**	7 dpi, 13 dpi, 17 dpi, and 7 dpc	7 dpi, 13 dpi, 17 dpi, and 7 dpc
14	**A137R (p11.5)**	7 dpi, 13 dpi, 17 dpi, and 7 dpc	13 dpi, 17 dpi, and 7 dpc
15	**A104R**	7 dpi, 13 dpi, 17 dpi, and 7 dpc	13 dpi, 17 dpi, and 7 dpc
16	**I73R**	7 dpi, 13 dpi, 17 dpi, and 7 dpc	13 dpi, 17 dpi, and 7 dpc
17	**C129R**	7 dpi, 13 dpi, 17 dpi, and 7 dpc	13 dpi, 17 dpi, and 7 dpc
18^*b*^	**MGF_110_12L**	7 dpi, 13 dpi, and 17 dpi	7 dpi, 13 dpi, 17 dpi, and 7 dpc
19	**B169L**	7 dpi, 13 dpi, 17 dpi, and 7 dpc	13 dpi, 17 dpi, and 7 dpc
20^*b*^	**G1340L**	7 dpi, 13 dpi, 17 dpi, and 7 dpc	13 dpi, 17 dpi, and 7 dpc
21	**F317L**	7 dpi, 13 dpi, 17 dpi, and 7 dpc	17 dpi and 7 dpc
22	**E120R (p14.5)**	17 dpi and 7 dpc	7 dpi, 13 dpi, 17 dpi, and 7 dpc
23	**E165R**	7 dpi, 13 dpi, 17 dpi, and 7 dpc	13 dpi, 17 dpi, and 7 dpc
24	**D1133L**	7 dpi, 13 dpi, 17 dpi, and 7 dpc	13 dpi, 17 dpi, and 7 dpc
25	**D117L (p17)**	7 dpi, 13 dpi, 17 dpi, and 7 dpc	7 dpi, 13 dpi, 17 dpi, and 7 dpc
26	K205R		7 dpi, 13 dpi, 17 dpi, and 7 dpc
27	E184L	13 dpi, 17 dpi, and 7 dpc	
28	B475L		7 dpi, 13 dpi, 17 dpi, and 7 dpc
29	A224L		7 dpi, 13 dpi, 17 dpi, and 7 dpc
30	CP123L	7 dpi, 13 dpi, 17 dpi, and 7 dpc	
31	DP238L		7 dpi, 13 dpi, 17 dpi, and 7 dpc
32	EP153R		7 dpi, 13 dpi, 17 dpi, and 7 dpc
33	MGF_300_4L		7 dpi, 13 dpi, 17 dpi, and 7 dpc
34	MGF_110_4L		7 dpi, 13 dpi, 17 dpi, and 7 dpc
35^*b*^	E66L		7 dpi, 13 dpi, 17 dpi, and 7 dpc
36^*b*^	A118R		7 dpi, 13 dpi, 17 dpi, and 7 dpc
37^*b*^	C62L		7 dpi, 13 dpi, 17 dpi, and 7 dpc
38^*b*^	B962L		7 dpi, 13 dpi, 17 dpi, and 7 dpc
39^*b*^	X69R	7 dpi, 13 dpi, and 17 dpi	
40^*b*^	MGF_110_7L	7 dpi, 13 dpi, 17 dpi, and 7 dpc	
41^*b*^	MGF_360_4L		7 dpi, 13 dpi, 17 dpi, and 7 dpc
42^*b*^	B438L (p49)	7 dpi, 13 dpi, 17 dpi, and 7 dpc	
43	O61R (p12)		7 dpi, 13 dpi, 17 dpi, and 7 dpc
44^*b*^	B385R	7 dpi, 13 dpi, 17 dpi, and 7 dpc	
45^*b*^	MGF_110_2L	7 dpi and 13 dpi	
46	D250R	7 dpi and 13 dpi	

^*a*^ The time points of positivity are shown. Proteins in bold are the common antigens of groups B and C.

^*b*^ Antigens newly found to be recognized by sera from pigs immunized with LAVs.

We next classified the 46 IgG-positive proteins into six categories according to their functions based on annotations from UniProt and reported literature (Table S2). Functional enrichment analysis (Table S3) revealed a significant overrepresentation of structural proteins in the 46 IgG-positive proteins with 1.56-fold enrichment, constituting 65.2% of the identified positive proteins. Furthermore, IgG responses tended to be induced preferentially by external structural proteins (proteins in the capsid, outer and inner envelopes of the virion) in the virion, with 2.61-fold enrichment. The overrepresentation of structural proteins in IgG-positive antigens may be related to higher expression levels of structural proteins than the non-structural ones (40).

### Temporal dynamics of IgG responses to ASFV antigens

**Fig 3:**
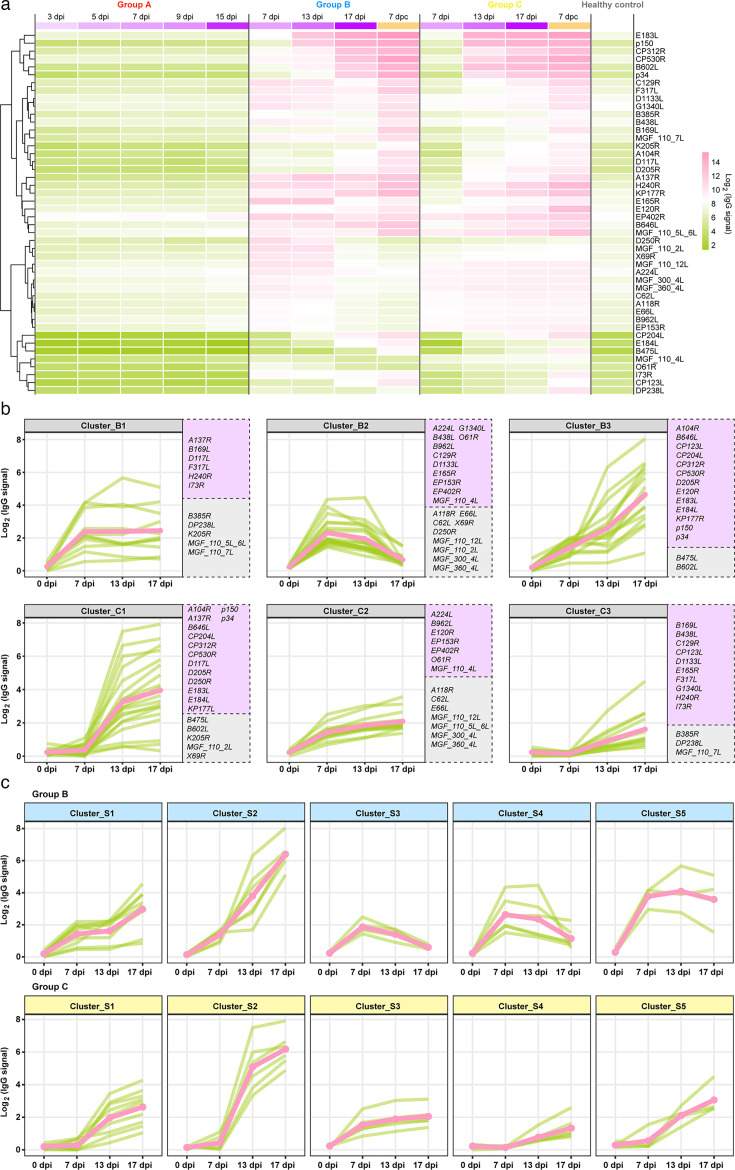
Temporal dynamics of the spectrum of IgG antibodies against ASFV antigens in infected pigs. (a) A timeline overview of the IgG responses to the 46 ASFV proteins in the three groups. Each rectangle indicates the average IgG signal against a specific ASFV protein (row) at a time point (column). (b) Distinct modes of the temporal dynamics of IgG responses in group B (upper panel) and group C (lower panel). Purple background, structural proteins; gray background, non-structural proteins. (c) Distinct modes of temporal dynamics of the IgG responses to 30 structural proteins, which are categorized into five clusters in groups B and C. In panels b and c, each light green line represents the IgG signal change against a specific protein, while the pink line reflects the dynamic mode based on the averaged IgG signals of all proteins in the corresponding cluster.

Next, we analyzed the temporal dynamics of IgG responses to ASFV proteins based on time-dependent antibody profiling data. An overview of the longitudinal IgG responses to the 46 ASFV antigens was generated with a heatmap (Fig. 3a). The correlation analysis results of IgG antibody spectra among individuals indicated low variability within groups in antibody responses after vaccination or challenge (Fig. S4). Pigs immunized with the two LAVs exhibited diverse dynamic changes in IgG responses to various viral proteins (some examples are shown in Fig. S5). In terms of seroconversion time, pigs exhibited early antibody responses to several viral proteins, including A224L, B475L, CP204L, and X69R, in both groups B and C at 7 dpi, whereas antibodies to proteins such as E184L and p150 became positive at 13 dpi. With respect to the peak time of antibody levels, IgG responses to some proteins, such as D250R, E165R, MGF_110_2L, and O61R, peaked at 7 dpi in group B, while for others, such as E183L and p150, the peaks occurred between 13 and 17 dpi in both groups B and C. In regard to the fluctuation of antibody levels, some proteins, including B475L, CP204L, CP312R, and K205R, displayed a sustained upward trend in the antibody level, whereas a delayed onset of the IgG response for certain proteins, such as B169L and B385R, manifested after challenge with the wild-type virulent strain.

To summarize the diverse dynamics of IgG responses, we conducted soft clustering in groups B and C separately (Fig. 3b). The 46 positive proteins within group B were categorized into three clusters based on the characteristics of changes in antibody levels over time. The antibody levels for Cluster_B1 (e.g., A137R and B385R) and B2 (e.g., A224L and A118R) exhibited the most remarkable increase rate from 0 to 7 dpi, with B1 remaining stable thereafter, while B2 showed a declining trend starting at 13 dpi. The antibody levels for proteins in Cluster_B3 (e.g., A104R and B475L) continued to increase during the immune process. Similarly, the positive proteins in group C were also categorized into three clusters, which are different from those in group B. The antibody levels for Cluster_C1-C3 continued to rise, with varying rates of increase between 7 and 17 dpi. The antibody levels for Cluster_C2 (e.g., A224L and A118R) exhibited the highest rate of increase from 0 to 7 dpi, followed by a gradual plateau from 7 dpi to 17 dpi. In contrast, Cluster_C1 (e.g., A104R and B475L) and C3 (e.g., B169L and B385R) showed an increase in the antibody level from 7 dpi on, with different rates of increase between the two clusters. Notably, the temporal dynamics of antibody responses to identical structural proteins can differ between groups B and C.

To further summarize the antibody dynamics of the IgG-positive structural proteins, we categorized them into five groups (S1–S5) based on their features in groups B and C (Table S4). As shown in Fig. 3c, the antibody responses of Clusters_S1 (e.g., A104R and B169L), S2 (e.g., CP204L and CP312R), and S5 (e.g., A137R and EP402R) continued to increase in both groups B and C, while the antibody responses of Clusters_S1 and S5 in group C were delayed. In contrast, antibody responses for Cluster_S3 (e.g., A224L and B962L) and S4 (e.g., B438L and C129L) rose and then fell over time in group B with a brief peak at 7 dpi but increased slowly in group C. The above features indicated that the antibody responses for Cluster_S1, S2, and S5 could be stably elicited by different attenuated ASFV mutants. However, the responses of Cluster_S3 and S4 showed different trends between groups B and C.

### Correlation analysis of IgG responses among the 46 positive proteins

**Fig 4:**
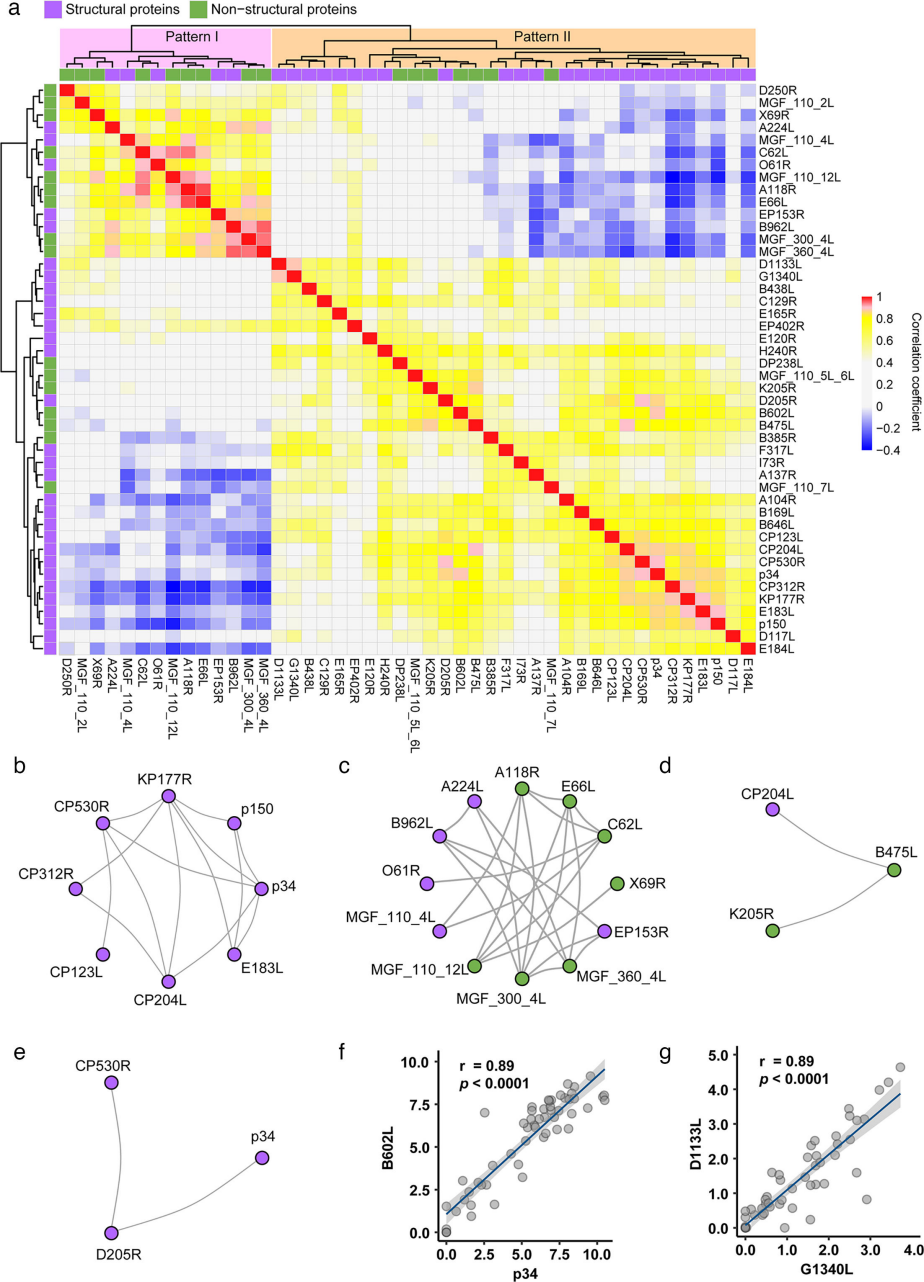
Correlation analysis of the IgG responses. (a) Heatmap displaying the Spearman correlation coefficients and clustering results among the 46 ASFV-specific IgG responses. (b-e) Network maps showing the protein subclusters that were highly correlated. (f and g) B602L-p34 (f) and D1133L-G1340L (g) correlations in IgG response. Each gray dot represents a serum sample.

In principle, when antigens are exposed to the immune system simultaneously, they stimulate it simultaneously and probably elicit correlated antibody responses, and vice versa. Therefore, for simple viruses, the antibody responses to early transcribed structural proteins and late transcribed non-structural proteins often exhibit different dynamic patterns (41). To explore the antibody response patterns of the complex ASFV, we analyzed the pairwise Spearman correlation coefficients among the 46 IgG-positive proteins based on their antibody level dynamics and visualized the results in groups B and C using a clustered heatmap (Fig. 4a). According to the results of unsupervised clustering, the 46 positive proteins fell into two main patterns (I and II). The antibody responses between proteins within the same pattern displayed varying degrees of positive correlations, while those between proteins of different patterns showed weak or no correlation. Interestingly, the antibody responses of non-structural proteins, such as MGF_110_7L, B385R, B475L, B602L, K205R, MGF_110_5L_6L, and DP238L, showed strong correlations with those of most structural proteins, while structural proteins, such as B962L, EP153R, O61R, MGF_110_4L, and A224L, did not correlate with most other structural proteins but showed strong correlations with some non-structural proteins in the antibody response. This result is unexpected if one considers the general notion of virus life cycles that the expression of structural and non-structural proteins is sequentially different between the two categories and synchronous within the same category, especially for structural proteins (42).

Specifically, several strong correlation networks of ASFV proteins with a coefficient of no less than 0.85 have been discovered. (i) The lower right section of the heatmap clustered some structural proteins exhibiting highly correlated antibody responses. Structural proteins, such as p150, p34, KP177R, CP530R, CP312R, CP123L, E183L, and CP204L, which were mainly categorized into Cluster_S1 and S2 (Fig. 3c), formed a highly correlated antibody response network I (Fig. 4b). These protein antigens may have similar spatiotemporal expression patterns. (ii) Another correlated network II (Fig. 4c), which was characterized by a different pattern of immune response, showed a slight negative correlation with the aforementioned structural proteins in the antibody response. (iii) The antibody response to the uncharacterized transmembrane protein B475L was strongly correlated with antibody responses to the multifunctional proteins CP204L and K205R (43), which regulate host interactions (Fig. 4d). (iv) The antibody response to the structural protein D205R was strongly correlated with those to two other structural proteins, p34 and CP530R (Fig. 4e). (v) B602L has a correlation coefficient of 0.89 with p34 (Fig. 4f). B602L participates in the hydrolysis of the polypeptide protein pp220 (44), a process that might simultaneously expose both B602L and the hydrolysis product p34 to the immune system and thus result in highly correlated antibody responses. (vi) The correlation coefficient between G1340L and D1133L is 0.89 (Fig. 4g). Both proteins are associated with viral transcription, with one predicted to be a helicase and the other a transcription factor (45, 46). Therefore, a high correlation of antibody responses may indicate similar expression dynamics, simultaneous immune exposure, and, thus, a possible function correlation of different ASFV proteins, though it is not always the case. These antibody response correlations revealed the characteristics of humoral immune responses induced by ASFV and might provide clues for studies on the functions and protein-protein interactions of ASFV proteins.

### Different dynamics of IgG responses between groups B and C

Pigs in groups B and C were infected with LAVs derived from the same parent strain but with different deletions of genes related to virulence or immune suppression. Therefore, it is interesting to ask whether there were differences in the antibody response dynamics between the two groups. To address this question, we conducted a comparative analysis of IgG antibody profiles at corresponding post-immunization time points (Fig. S6). Notably, at 7 dpi, the IgG responses to the 30 proteins in group B were significantly greater than those in group C. However, at 13 dpi, only three proteins in group B maintained significantly stronger antibody responses than did those in group C. Remarkably, at 17 dpi, there were no discernible differences in the antibody responses between groups B and C. The dynamics comparison revealed obviously delayed IgG production in group C compared with that in group B. The pivotal disparities in antibody responses between groups B and C primarily manifested at 7 dpi. Afterward, the two groups became increasingly similar in terms of IgG responses, with no significant differences before the viral challenge (19 dpi).

To characterize the antibody responses in terms of seroconversion time in groups B and C, functional enrichment analysis was conducted on proteins eliciting earlier (at 7 dpi) and later (later than 7 dpi) antibody responses (Table S5). Structural proteins were enriched in group B’s early seroconversion proteins and group C’s late seroconversion proteins. In contrast, transmembrane proteins were enriched in the early seroconversion proteins of group C. This result indicated that the delayed antibody production in group C mainly targeted structural proteins.

The above results comprehensively delineated the differences in antibody dynamics following immunization with the two different LAVs, which may shed light on the mechanisms underlying antibody production, virus-pig interactions, and immune protection conferred by attenuated viruses.

### Ranking of ASFV B-cell antigens with regard to vaccine design

**Fig 5:**
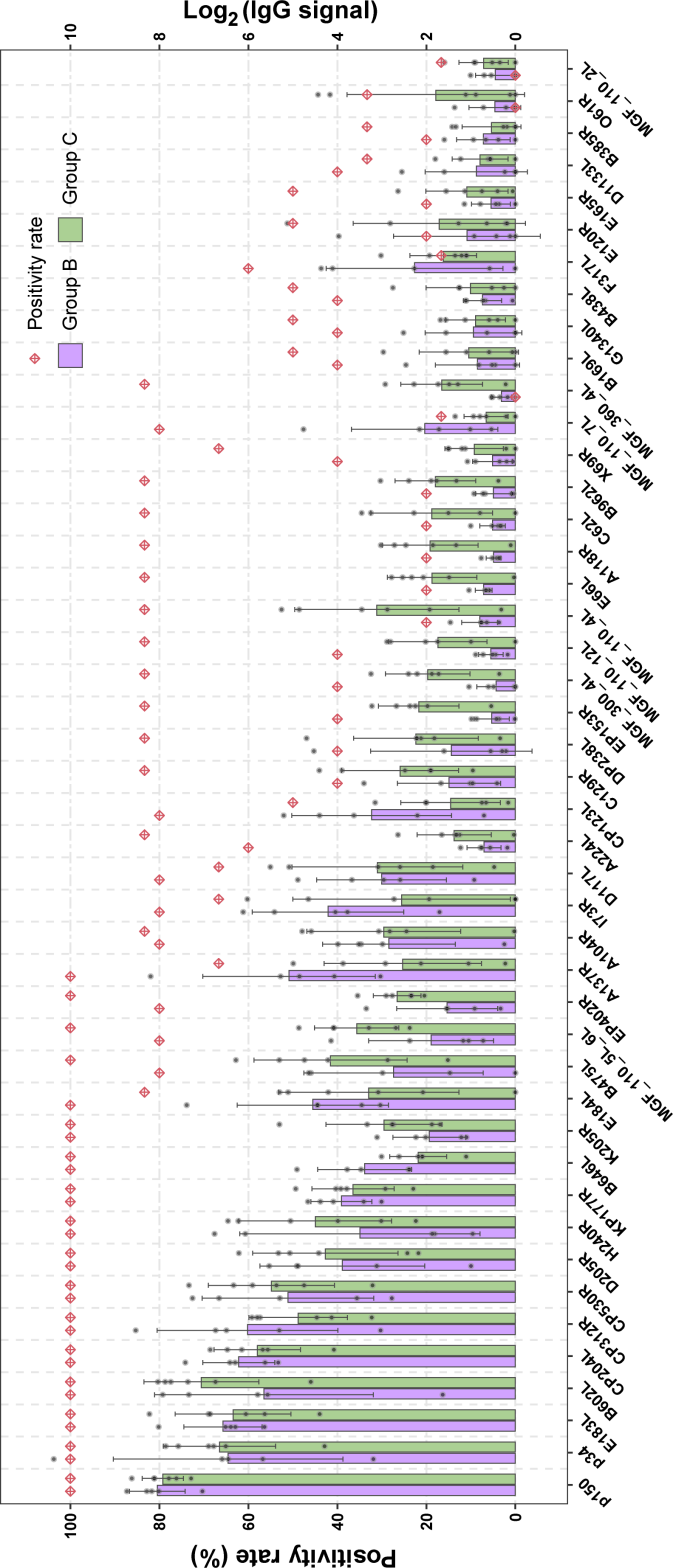
Average antibody levels and positivity rates of the identified proteins in groups B and C based on the data of 17 dpi. A red diamond indicates the IgG-positivity rate of an ASFV protein among the pigs in group B or C. Histograms show the signal intensities (Log_2_ [IgG signal]) of reactive antigens (mean value ± SD) with gray points representing the signal intensities of individual pigs. Proteins are sorted in a descending order based on their average positivity rates and average antibody signal intensities.

**Fig 6:**
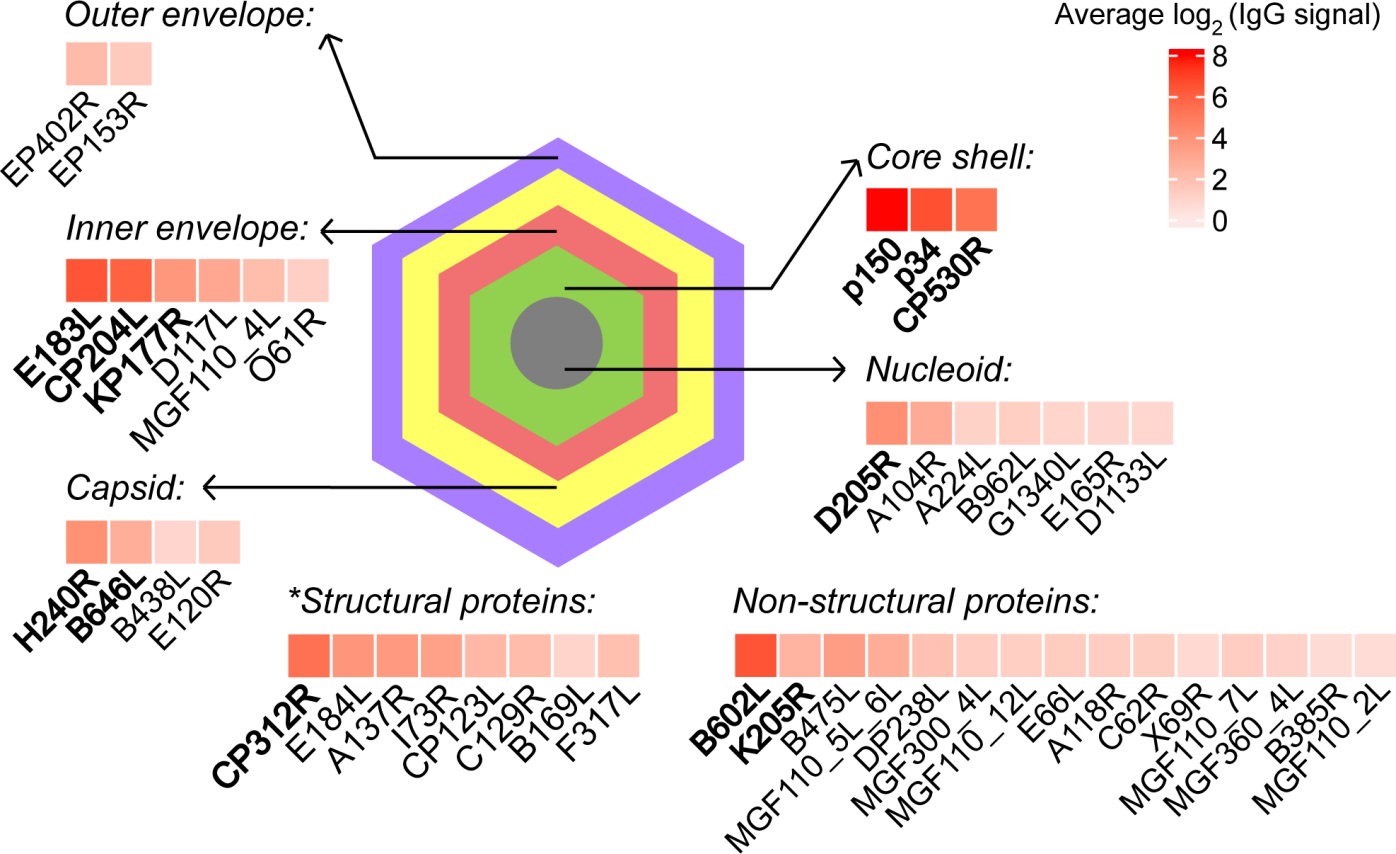
ASFV atlas of the identified B-cell antigens. The protein locations in the ASFV virion were determined according to previous reports (7, 8, 48). The color in the box represents the average antibody level of the protein below at 17 dpi in groups B and C. Bold font indicates a 100% positivity rate in both groups B and C at 17 dpi. The asterisk indicates structural proteins whose subviral locations have not yet been determined.

Although an effective vaccine for ASFV needs a combination of multiple antigens, a ranking of the B-cell antigens in terms of positivity rates and antibody levels would be helpful to guide the design of such combinations. It is noteworthy that, apart from a few proteins, the antibody responses to most proteins did not significantly change after challenge with the wild-type virulent strain (17 dpi vs seven dpc in Fig. S7). So, we mapped the antibody spectrum at 17 dpi and found 45 proteins exhibiting high positivity rates and antibody levels (Fig. 5). Among the 12 proteins with the highest percentages of positivity (100%) in both groups B and C, 10 are structural proteins belonging to clusters S1, S2, and S5 (Table S4), which induced stable IgG production in both LAVs (Fig. 3c), and the remaining two are non-structural proteins, which also demonstrated stable or increasing levels of IgG. Interestingly, the reported neutralizing antigens E183L (14, 19), CP204L (14, 19), and B646L (14, 47) are among these 12 proteins. Another reported neutralizing antigen, EP402R (14, 20), also exhibited a high positivity rate (≥80%) and antibody levels (Fig. 5). More importantly, we identified 12 new B-cell antigens induced by LAVs. Among these new antigens, MGF_110_7L and B385R induced stable antibody responses, while MGF_300_4L, MGF_110_12L, E66L, A118R, C62L, B962L, X69R, MGF_360_4L, G1340L, and MGF_110_2L induced plastic antibody responses. As a summary of the 45 B-cell antigens, Fig. 6 illustrates the locations of the structural proteins in the ASFV virion, with the non-structural proteins listed underneath. The B-cell antigen repertoire of ASFV based on LAV-protected pigs would help design combinatorial-antigen vaccines.

## DISCUSSION

A great deal of effort has been devoted to the study of B-cell antigens, especially structural proteins, to deepen the understanding of ASFV virology, search for detection biomarkers of ASFV, and discover potentially protective B-cell antigens (39, 49–51). However, progress has been slow due to the lack of a systematic study tool, and no comprehensive understanding has been established. ASFV B-cell antigens have been reported only sporadically. In this study, we created a microarray containing 160 ASFV proteins (covering more than 95% of the whole ASFV proteome), including 67 structural and 93 non-structural proteins. The microarray enables a more comprehensive, profound, and unbiased analysis of ASFV humoral responses.

Based on the microarray data, the humoral immunoproteome of ASFV was established using serum samples from pigs vaccinated with two LAVs that conferred complete protection. The humoral immunoproteome consisted of 46 positive proteins, 12 of which are newly recognized B-cell antigens and 34 of which have been reported in the literature. Among the reported antigens, antibodies to some proteins were detected in pig sera vaccinated with other attenuated viruses or in ASF-positive pig sera, while the immunogenicity of others was validated by immunizing animals through various delivery methods (12, 49, 52). The seven proteins showing high immunogenicity with 100% positivity rates in our microarray results (Fig. 4), p150 (53, 54), p34 (39, 49, 53, 54), E183L (39, 49–51), B602L (49–51), CP204L (39, 49–51), CP312R (49–51), and CP530R (39, 55), were reported in previous studies. Recently, Ferreira et al. reported that the level of total IgG antibodies against A104R was steadily elevated from 7 to 20 dpi (56), which aligns closely with the antibody dynamics observed in our groups B and C swine cohorts (Fig. 3b). These reported results support the credibility of our microarray-based serum analysis results. The microarray can be applied for comparative analysis of antigen spectra from different LAVs, from which we may identify the common antigens that could be potential protective candidates. On the other hand, some ASFV B-cell antigens identified in pigs immunized with LAVs in previous studies, such as K78R (49–51), I177L (49), E199L (49), and H171R (39, 49), were not included among our 46 IgG-positive proteins. This might be attributed to differences in attenuated strains, heterogeneity in the pigs’ immune status, or the possibility that these proteins may not have folded correctly and thus generated false negative results. An additional limitation is that the proteome on our microarray may not completely match that of a given ASFV strain, considering the high diversity of ASFV strains and mutants. Therefore, one should take the proteome difference into account when extending our findings to other strains or using the microarray to analyze samples resulting from the infection of other strains.

Compared with group B pigs, group C pigs showed delayed antibody responses, although they were infected by two LAVs, with only minor differences in deleting two or three different genes. Group B pigs were immunized with the attenuated ASFV-GS-Δ18R/NL/UK mutant, and group C pigs were immunized with the ASFV-GS-ΔMGF110/360-9L mutant. At 7 dpi, antibody responses to 30 viral proteins in group B were significantly greater than those in group C. However, these antibody responses became similar between groups B and C at 17 dpi, which can be explained by the fact that both mutants were derived from the same strain and have nearly identical antigenic contents. Transcriptomic results from LAV-infected cell models showed that ASFV-GS-ΔMGF110/360-9L induced more robust innate immune responses and proinflammatory responses than did the parent strain (36). At the animal level, individual pigs immunized with ASFV-GS-Δ18R/NL/UK showed detectable virus in the serum at 2 dpi (35), while pigs immunized with ASFV-GS-ΔMGF110/360-9L showed detectable virus until 5–7 dpi (36). These results suggested that pigs immunized with ASFV-GS-ΔMGF110/360-9L exhibited early innate immune responses that suppressed early virus replication, delaying the time at which the progeny viruses entered immune system surveillance. This delay leads to later exposure to the adaptive immune system, resulting in delayed antibody responses.

The LAVs conferred protection in both groups B and C, though the mechanisms are not well understood. There are reports on positive correlations between the antibody titers and protection effects (16, 17). However, a popular view is that both humoral and cellular immune responses are vital to protection (57, 58). Our study provides a systematic tool to address the humoral immunity part. If protective B-cell antigens of ASFV did exist, they should be part of the B-cell antigen repertoire (Table 1). In a study by Netherton et al., a pool of eight antigens delivered by viral vectors provided 100% protection against a genotype I European ASFV strain (13). Five of the eight proteins, B602L, B646L, CP204L, E183L, and F317L, elicited detectable antibodies after immunization or challenge. These five proteins are included in the 25 common antigens identified in our study. In a more recent study, antibody responses to structural proteins of ASFV were investigated in pigs immunized with an LAV (genotype II) that conferred complete protection. Significant antibody responses to 16 ASFV proteins were identified (39), 12 of which were among the 46 proteins identified in our study. However, a strong antibody response does not readily imply a better protective effect. Anti-ASFV antibodies can even mediate enhancement of diseases through yet unknown mechanisms (59). For developing subunit vaccines, establishing the ASFV reactome is the first step of many. It is critical in future work to figure out protective antigens from the reactome and the antigen combinations that provide efficient protection. More profiling data of humoral immune responses from protected pigs by LAVs of different strains and genotypes may help to concentrate the B-cell antigen pool that is potentially associated with (cross) protection, which can ease the testing of protective effects of ASFV antigens and antigen combinations. Also, microarray-enabled systematic comparisons of responses in animals that have enhanced disease after immunization and challenge might provide helpful information on disease progression associated with specific antibody responses.

In conclusion, we have constructed a proteome microarray of ASFV, used it to scan antibodies against nearly all viral proteins in infected pigs, and established the B-cell antigen repertoire of ASFV. We have also revealed the temporal dynamics of antibody responses and identified B-cell antigens that have not been previously reported. These findings provide systematic insights into humoral immune responses against the ASFV proteome after acute infection, LAV immunization, and post-vaccination challenge. The established B-cell antigen repertoire will be useful for developing safe and effective subunit or vectored vaccines. The proteome microarray offers a high-throughput platform for understanding ASFV immunology and pathogenicity and will contribute to effective and fast control of this deadly virus.

## MATERIALS AND METHODS

### Construction of expression vectors

The nucleotide sequence of the ASFV-SY18 isolate was downloaded from GenBank (accession number: MH766894.1). The amino acid sequences of the predicted ASFV ORF of no less than 50 aa were converted into *Saccharomyces cerevisiae* codon-optimized gene sequences. The sequences of 168 optimized genes were synthesized by Sangon Biotech (Shanghai, China). Then, the sequences were cloned and inserted into two expression vectors, pEGH-A (34) and pESC-URA (Stratagene, California, USA), for protein expression, and the expression vector with the highest expression was selected to express the protein. An ASFV protein-encoding sequence was cloned and inserted into the pEGH-A vector at the BamHI and NotI restriction sites (Thermo Fisher Scientific, Waltham, MA, USA), positioning the GST tag sequence at the N terminus of the recombinant fusion protein to generate pEGH-A-GST-ASFV. The encoding sequence of a fusion protein was amplified by PCR using pEGH-A-GST-ASFV as the template. The sequences were then inserted into the pESC-Ura vector at the BamHI and NotI restriction sites (Thermo Fisher Scientific, Waltham, MA, USA) under the control of the Gal1 promoter to generate the vector pESC-Ura-GST-ASFV. The expression vectors were constructed using a ClonExpress II One Step Cloning Kit (Vazyme, Nanjing, China). All constructs were confirmed by DNA sequencing (Sangon Biotech, Shanghai, China).

### Microarray protein preparation

The expression vectors were transformed into the *Saccharomyces cerevisiae* Y258 strain by using the LiAC/SS carrier DNA/polyethylene glycol (PEG) method to construct the transformants (60). The *Saccharomyces cerevisiae* Y258 transformants were spread onto plates of a selection of uracil (Ura)-deficient synthethic complete (SC) culture media (SC minus Ura) supplemented with glucose and incubated at 30°C for 2 days. The individual clones were amplified on liquid SC minus Ura media supplemented with glucose at 90 rpm and 30°C for 2 days. The *Saccharomyces cerevisiae* stock culture was transferred at a ratio of 1:1,500 into a larger volume of SC minus Ura medium containing raffinose as the carbon source for amplification as well as for the elimination of glucose-mediated repression of recombinant protein production at 90 rpm and 30°C for 16 h (OD_600_ = 0.6–0.8). Then, a final concentration of 2% galactose was added to the culture medium to induce the expression of the recombinant GST-tagged ASFV proteins at 90 rpm and 30°C for 6 h. Then, the centrifugation-harvested cells were mechanically crushed using zirconium-bead-based agitation, and the GST-tagged target proteins were affinity purified from the supernatant of the mixture using glutathione-agarose beads (Senhui Microsphere Technology, Suzhou, China). Next, the proteins were concentrated using ultracentrifugal filters (Merck Millipore, USA) with corresponding molecular weight cutoffs. Finally, the purified proteins were analyzed by SDS-PAGE followed by western blotting using an anti-GST antibody (Abcam, UK, Cat# ab19256) and silver staining.

### Protein microarray fabrication

The 160-protein library (5 µg/mL for each protein) was used to prepare the protein microarray following a previously described procedure (61). Briefly, using a Super Marathon printer (Arrayjet, UK), identical protein arrays in a 2 × 7 subarray format were generated by printing affinity-purified 160 ASFV proteins, accompanied by negative (BSA and GST) and positive controls (anti-swine IgG [Novus Biologicals, USA, Cat# NBP1-97054], IgM [Novus Biologicals, USA, Cat# NBP1-96788], and IgA [Alpha Diagnostic International, USA, Cat# 20017-4-1]) and land markers, in triplicate, on PATH Protein Microarray Slides (GraceBio-Labs, Oregon, USA). The microarrays obtained were kept frozen at a temperature of −80°C until use. To monitor the proteins contained in each spot, we probed antibodies against the N-terminal GST tags engineered into each protein.

### Pig infection and vaccination experiments

This study was performed in strict accordance with the recommendations in the Guide for the Care and Use of Laboratory Animals of the Ministry of Science and Technology of the People’s Republic of China. The procedures were performed according to the Guiding Principles for Biomedical Research Involving Animals.

Experiments involving live ASFV were conducted in biosafety level 3 facilities at LVRI of CAAS and were approved by the Ministry of Agriculture and Rural Affairs. Farm-raised Large White-Duroc crossbred pigs were obtained from a licensed livestock farm. Each pig was confirmed to be antigenically and serologically negative for ASFV and tested negative for porcine respiratory and reproductive syndrome, pseudorabies virus, and classical swine fever.

Pigs weighing 30–40 kg were infected with the highly virulent ASFV CN/GS/2018 strain (1 HAD_50_) or the genetically engineered attenuated ASFV/GS mutant (10^4^ HAD_50_). Furthermore, pigs that survived beyond the observation period (17 dpi) after being infected with the genetically engineered attenuated mutant were challenged with the parental strain (10^2^ HAD_50_) to assess the protective effect. A challenge experiment was conducted at 19 dpi. Serum samples were collected from all pigs before and after infection/vaccination until death or the endpoint of treatment.

### Microarray-based serum analysis

Serum profiling on a protein microarray was carried out as previously described (62). In brief, the ASFV proteome microarrays were initially treated with 3% fat-free milk for 1 h at room temperature and then rinsed three times with PBST (phosphate buffered saline [PBS] containing 0.1% Tween 20). Individual chambers for the 14 identical subarrays were created by mounting a 14-chamber rubber gasket onto each slide. Then, the slides were incubated with 200 × diluted pig sera for 3 h at room temperature, followed by washing three times in PBST. Afterward, the microarrays were incubated with either rabbit anti-swine IgG antibody (Novus Biologicals, USA, Cat# NBP1-72784) followed by Cy3-AffiniPure donkey anti-rabbit IgG (H + L) (Jackson ImmunoResearch, PA, USA, Cat# 711-165-152) for IgG detection, or mouse anti-swine IgM antibody (AbD Serotec, Germany, Cat# MCA637GA) followed by Alexa Fluor 647 AffiniPure Donkey Anti-Mouse IgG (H + L) (Jackson ImmunoResearch, PA, USA, Cat# 715-605-150) for IgM detection. After being washed three times and air-dried through brief centrifugation, the slides were scanned using a Genepix 4200A scanner (Molecular Devices, CA, USA).

### Protein array data processing

The median foreground and background fluorescence intensities were measured for each spot on the protein microarrays using GenePix Pro 6.0 (Molecular Devices, CA, USA). The fluorescence intensity was calculated as the median foreground minus the median background for each spot, followed by averaging the triplicate spots for each of the 160 ASFV proteins. By using the data of the positive control block in each slide, the normalization factor was calculated for each protein in each microarray. For each slide, all the fluorescence intensities were normalized by multiplying the corresponding normalization factors. The threshold was set as the mean signal of the healthy control group plus three times the SD. In the experimental groups, either infected or vaccinated, a viral protein was considered IgG- or IgM-positive if its fluorescence intensity on the ASFV microarray in at least one pig serum sample exceeded the threshold. To analyze ASFV proteins eliciting a relatively strong antibody response, proteins with an average fluorescence intensity of less than 300 in the group were filtered out. The positivity rate of an ASFV protein was the percentage of antibody-positive pigs among the total number of live pigs in each group at each time point. Antibody levels are presented as log_2_ (fluorescence intensity) values.

### Quantification and statistical analysis

Data analysis and visualization were performed using R software 4.1.2. To evaluate the quality of the generated data, we perform unsupervised clustering of the data and visualize all the features in a lower dimension by using the “umap” package. A Venn diagram illustrating positive proteins between groups was generated using the “VennDiagram” package. We used the “pheatmap” package to construct a heatmap of the antibody responses to the 46 positive ASFV proteins obtained from all the serum samples. Classification of the temporal dynamic behaviors of the antibody response in groups B or C was conducted by soft clustering using the “Mfuzz” package (63) and visualized using the “ggplot2” package. Before being utilized for analysis, the log_2_-transformed fluorescence intensity of each positive protein from groups B and C was first subtracted by that from the healthy control group, which was used as the background. Since there was no difference in the antibody profiles between the healthy control group and group B at 0 dpi, the antibody signal from group B at 0 dpi was also used as representative data for 0 dpi in group C. To group the temporal dynamics of antibody responses to structural proteins based on the antibody level dynamics in groups B and C simultaneously, cluster analysis was performed by using the “pheatmap” package. Then, we drew line graphs depicting the antibody responses of proteins in each cluster over time based on the classification results using the “ggplot2” package. Fisher’s exact test was used for category enrichment analysis to help functionally characterize the positive proteins. The correlations of samples without a normal distribution were represented by the Spearman correlation coefficient. A pairwise correlation analysis was conducted with the cor() function. Proteins with a correlation coefficient of antibody response greater than 0.85 are shown. Proteins exhibiting network relatedness were visualized using network graphs, while proteins with individually high correlations were presented using scatter plots. Comparisons of antibody responses between different groups or time points were conducted by the “limma” package (64). Proteins were considered significantly different in the antibody signature if they had a false discovery rate (FDR) < 0.05 and an absolute log_2_(FC) > 0.5 between the groups.

## Data Availability

The raw array data have been deposited in the protein microarray database (http://www.proteinmicroarray.cn/) under the accession number PMDE266. The authors confirm that the data supporting the findings of this study are available within the article and/or its supplemental material.

## SUPPLEMENTAL MATERIAL

Supplemental material jvi.00478-25-s0001.docx Figures S1 to S7 and Tables S1 to S5.

ASM does not own the copyrights to Supplemental Material that may be linked to, or accessed through, an article. The authors have granted ASM a non-exclusive, world-wide license to publish the Supplemental Material files. Please contact the corresponding author directly for reuse.
