# Supplementary material for: An African swine fever virus-specific antibody reactome reveals antigens as potential candidates for vaccine development
Source: J Virol. 2025 Aug 14;99(9):e00478-25. doi: 10.1128/jvi.00478-25 (PMC12455989; doi:10.1128/jvi.00478-25)
Supplement: Supplemental material — Figures S1 to S7 and Tables S1 to S5. [file jvi.00478-25-s0001.docx]

Supporting Information for

**An African Swine Fever Virus-Specific Antibody Reactome Reveals Antigens as Potential Candidates for Vaccine Development**

Songxin Guo^1,2#^, Yi Ru^3#^, Hui Zhang^1^, Junbiao Xue^4^, Huanan Liu^3^, Dong Men^1,5^, Zongqiang Cui^1,2^, Chaochao Shen^3^, Hong Tian^3^, Chun Ma^1,2^, Jun Gong^1,2^, Jintian Xu^1,2^, Dianbing Wang^2,6^, Rui Gong^2,6^, Xiaowei Zhang^1^, Heng Rong^1,2^, Yan-Yi Wang^1,2^, Chenli Liu^7^, Zhuojun Dai^7^, Shengce Tao^4^*, Jiaoyu Deng^1,2^*, Haixue Zheng^3^*, Feng Li^1,2^*, Xian-En Zhang^6,8^*

^1^Key Laboratory of Virology and Biosafety, Wuhan Institute of Virology, Chinese Academy of Sciences, Wuhan, China.

^2^University of Chinese Academy of Sciences, Beijing, China.

^3^State Key Laboratory for Animal Disease Control and Prevention, College of Veterinary Medicine, Lanzhou University, Lanzhou Veterinary Research Institute, Chinese Academy of Agricultural Sciences, Lanzhou, China.

^4^Key Laboratory of Systems Biomedicine (Ministry of Education), Shanghai Center for Systems Biomedicine, Shanghai Jiao Tong University, Shanghai, China.

^5^Guangzhou Laboratory, Guangzhou, China.

^6^National Laboratory of Biomacromolecules, Institute of Biophysics, Chinese Academy of Sciences, Beijing, China.

^7^Institute of Synthetic Biology, Shenzhen Institute of Advanced Technology, Chinese Academy of Sciences, Shenzhen, China.

^8^Faculty of Synthetic Biology, Shenzhen University of Advanced Technology, Shenzhen, China.

*^#^*These authors contributed equally to this work.

*Email: zhangxe@ibp.ac.cn; [fli@wh.iov.cn](mailto:fli@wh.iov.cn); haixuezheng@163.com; [dengjy@wh.iov.cn](mailto:dengjy@wh.iov.cn); [taosc@sjtu.edu.cn](mailto:taosc@sjtu.edu.cn).

**This PDF file includes:**

Figs. S1 to S7

Tables S1 to S5


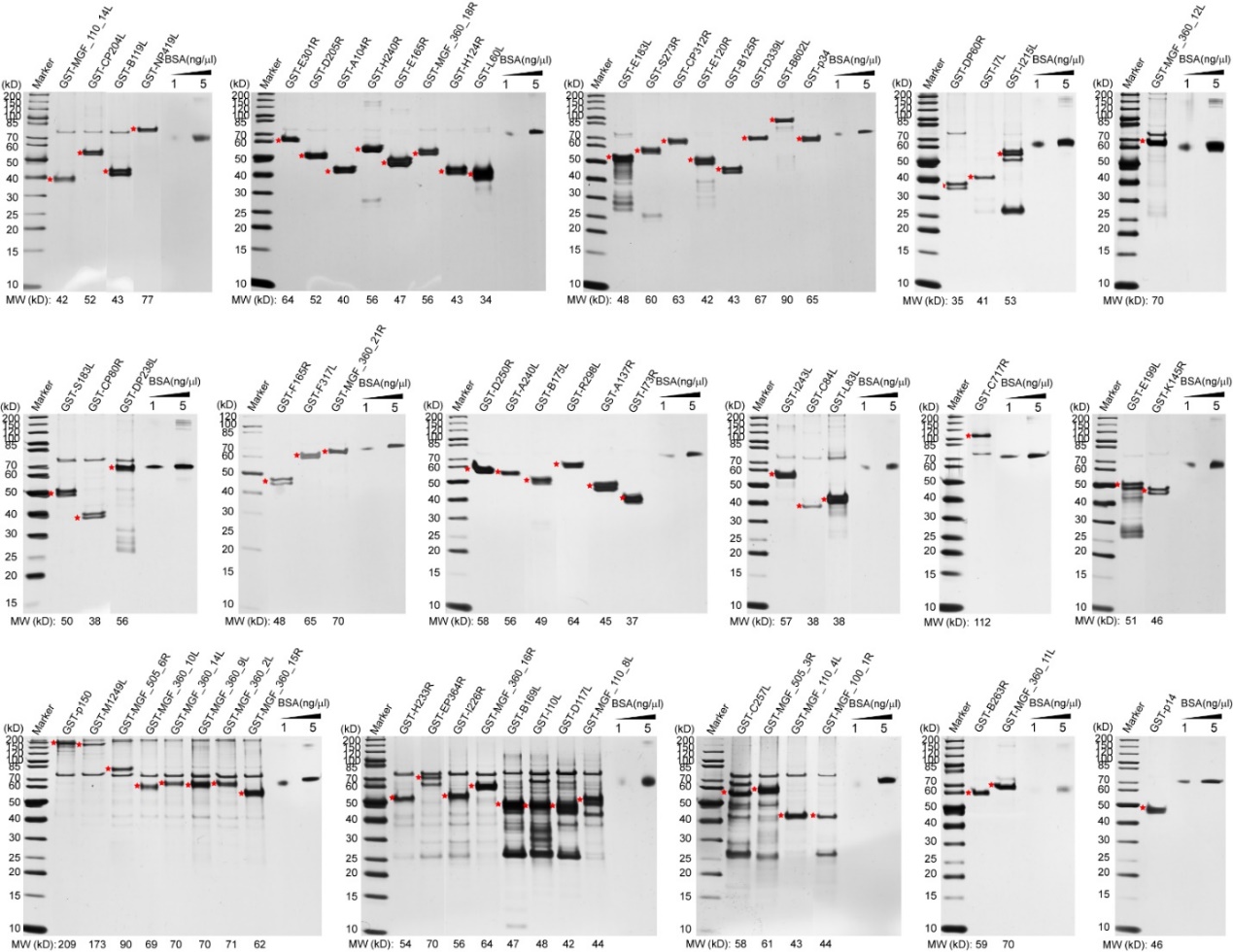


Fig. S1. Construction of the 160-protein library of ASFV. Representative SDS‒PAGE results of purified ASFV proteins. Each red asterisk marks the band of the expected size for a given protein.


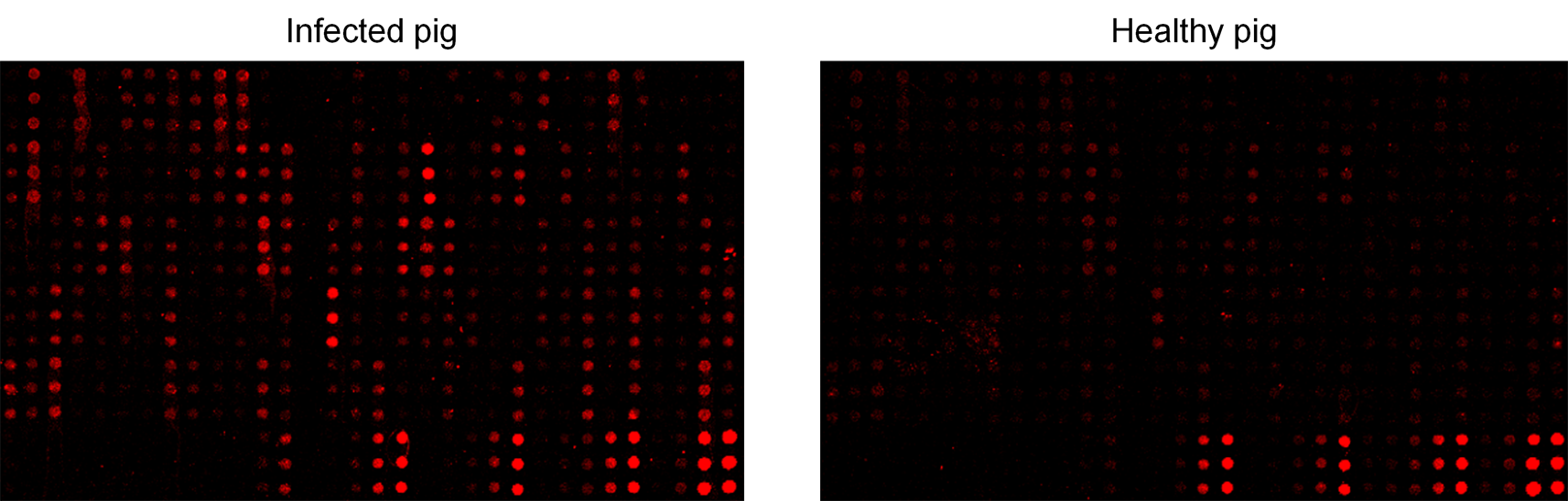


Fig. S2. Representative subarrays probed with sera from an ASFV-infected pig and a healthy pig. The IgM signals are shown in red.


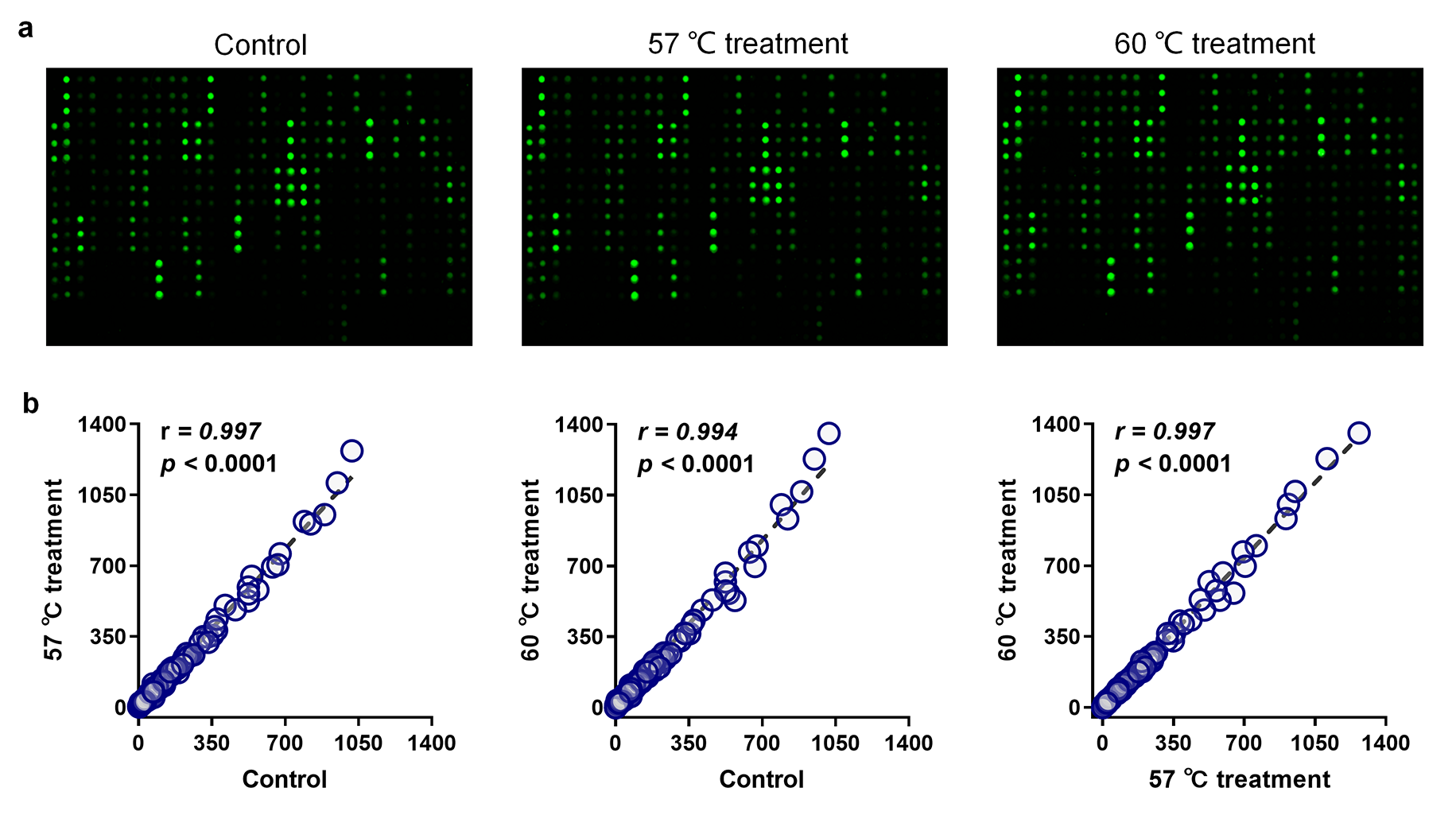


Fig. S3. Serum inactivation did not affect antibody detection. (a) Protein microarray analyses of one serum sample from an ASF pig after various treatments, including no treatment, incubation at 57 ^o^C for 1 h, and incubation at 60 ^o^C for 40 min. (b) Correlations of the IgG responses among the 57 ^o^C-treated, 60 ^o^C-treated, and untreated (control) samples.


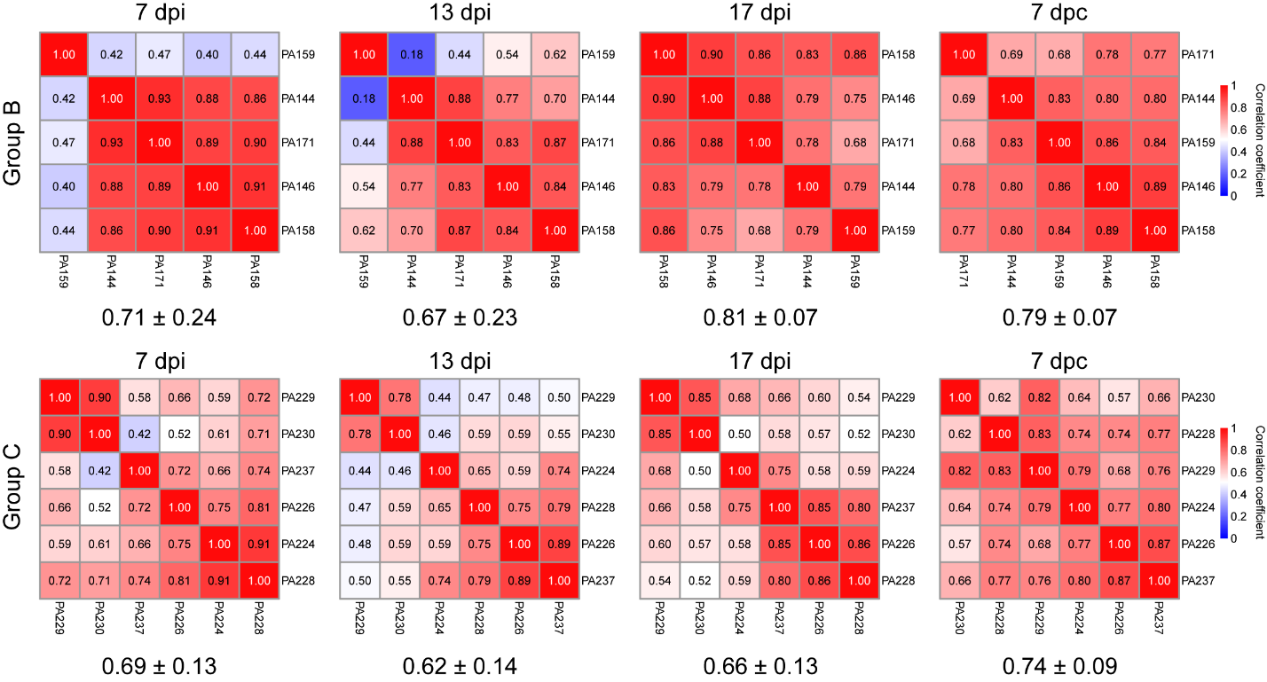


Fig. S4. Low variation in the IgG spectrum among individual pigs after immunization with the same attenuated vaccine and after challenge. Spearman correlation matrices of IgG responses among individual pigs at the indicated dpi or dpc in groups B and C are shown. The number under each matrix indicates the Spearman correlation coefficient (mean value ± SD).


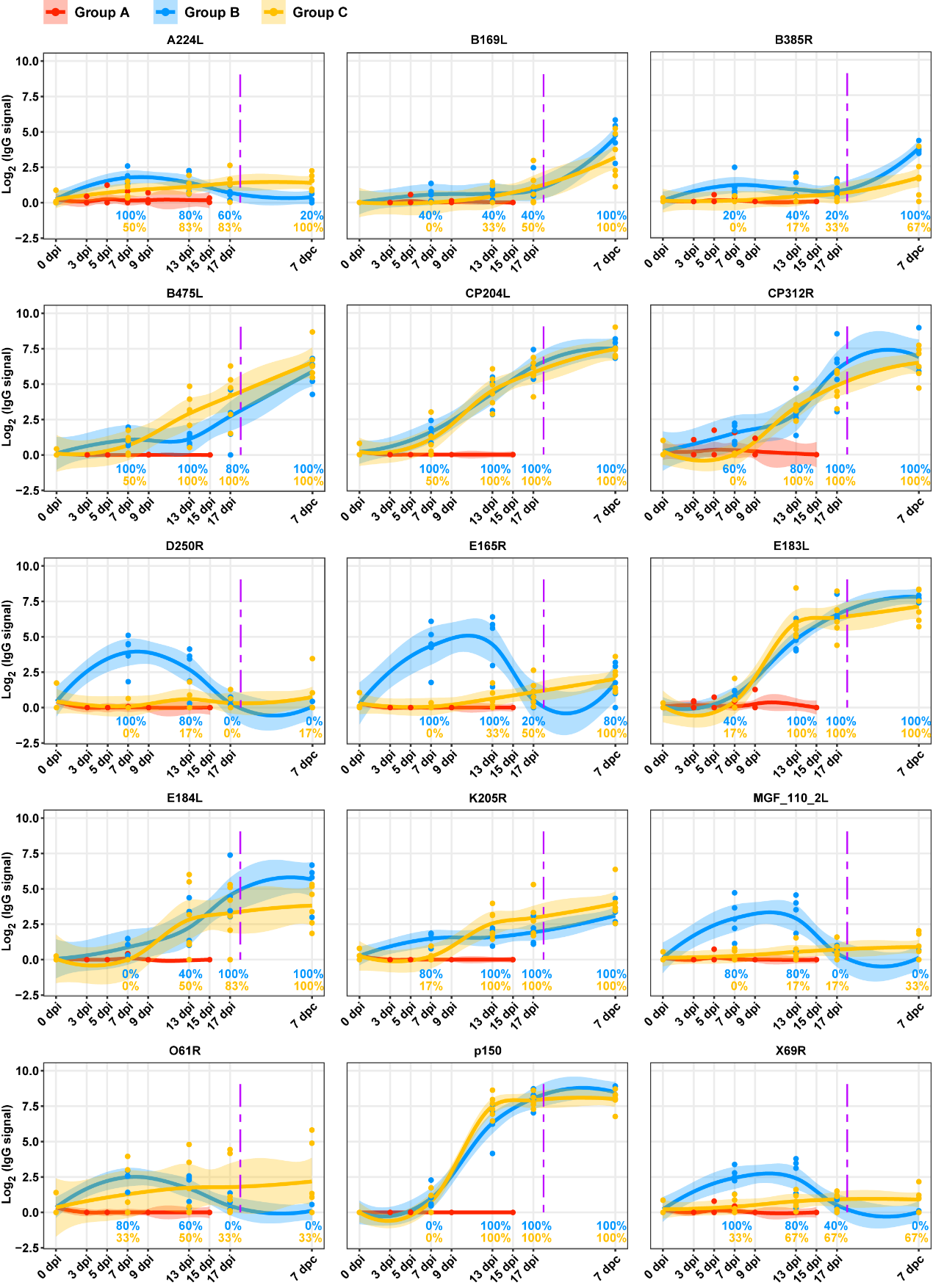


Fig. S5. Dynamics of representative IgG responses to ASFV proteins in the three groups. Each dot represents the IgG signal from an individual pig. Smooth curves, which were generated by locally weighted linear regression, show the IgG signal dynamics. The shading beneath the curve represents the 95% confidence interval. The dashed purple lines indicate the challenge time. The positivity rates of the antibody response in groups B (blue) and C (yellow) are indicated.


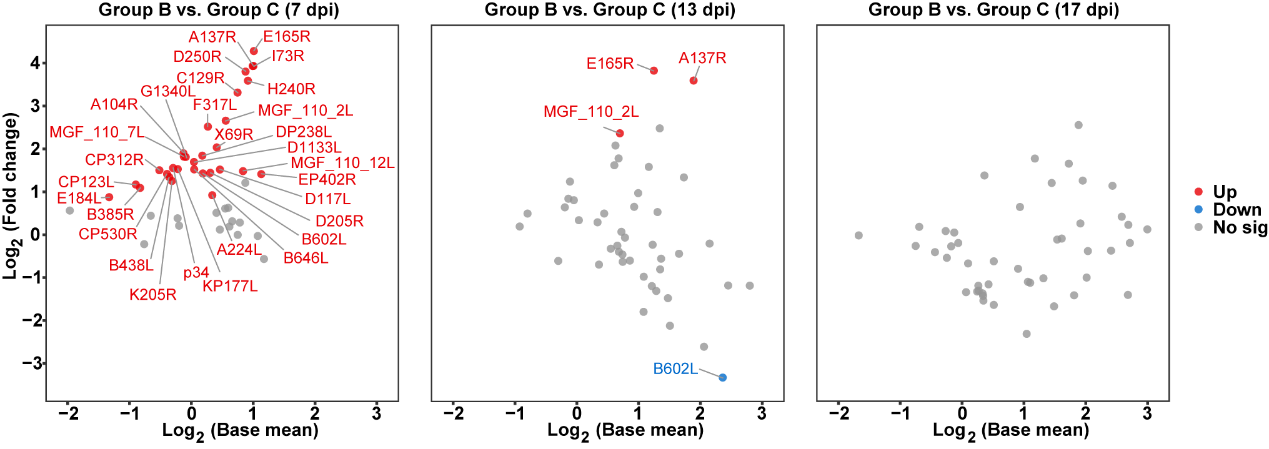


Fig. S6. Comparison of the IgG spectra between groups B and C over time from 7 dpi to 17 dpi. MA plots show the log_2_ (fold change) (y-axis) (M-values, the log_2_ of the ratio of the IgG signal for each protein between groups B and C at the indicated time points) against the log_2_ average (x-axis) (A-values, the average IgG signal for each protein across the sample). Each dot represents one protein.


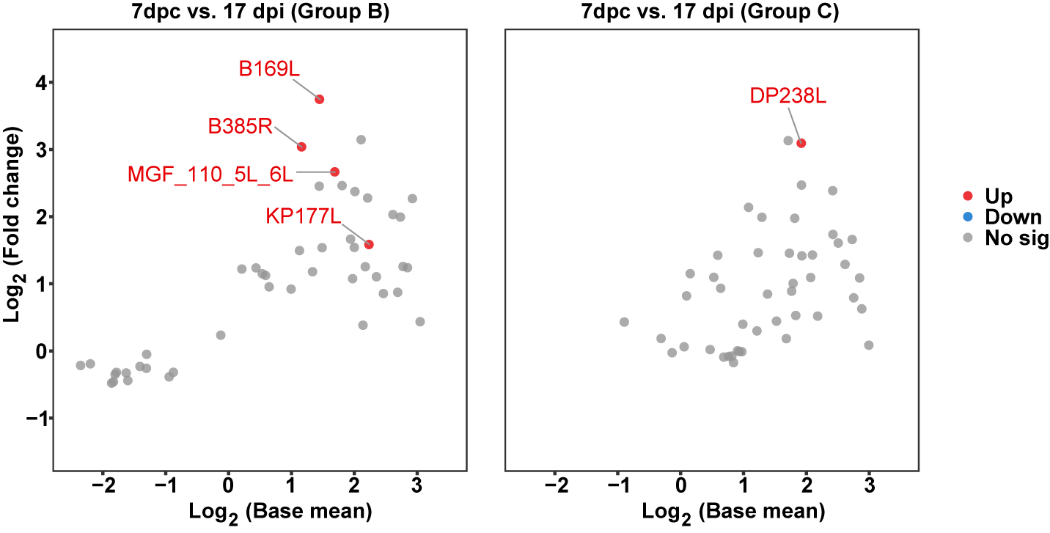


Fig. S7. Comparison of the IgG spectra between 7 dpc and 17 dpi in groups B and C. MA plots show the log_2_ (fold change) (y-axis) (M-values, the log_2_ of the ratio of the IgG signal for each protein between 7 dpc and 17 dpi in the indicated groups) against the log_2_ average (x-axis) (A-values, the average IgG signal for each protein across the sample). Each dot represents one protein.

Table S1. IgM-positive ASFV proteins identified in groups B and C. The time points of positivity are shown. Proteins in bold are the common antigens of groups B and C.

|  | **Protein** | **Group B** | **Group C** |
| --- | --- | --- | --- |
| 1 | **CP312R** | 7 dpi, 13 dpi, 17 dpi and 7 dpc | 13 dpi, 17 dpi and 7 dpc |
| 2 | **E183L (p54)** | 7 dpi, 13 dpi, 17 dpi and 7 dpc | 13 dpi, 17 dpi and 7 dpc |
| 3 | **p150** | 7 dpi, 13 dpi, 17 dpi and 7 dpc | 13 dpi, 17 dpi and 7 dpc |
| 4 | **E165R** | 7 dpi, 13 dpi, and 7 dpc | 17 dpi and 7 dpc |
| 5 | **EP402R (CD2v)** | 7 dpi, 13 dpi and 17 dpi | 17 dpi and 7 dpc |
| 6 | **CP530R (pp62)** | 7 dpi, 13 dpi, 17 dpi and 7 dpc | 13 dpi, 17 dpi and 7 dpc |
| 7 | **B602L** | 7 dpi, 13 dpi, 17 dpi and 7 dpc | 13 dpi, 17 dpi and 7 dpc |
| 8 | KP177R (p22) | 7 dpi, 13 dpi, 17 dpi and 7 dpc |  |
| 9 | H240R | 7 dpi, 13 dpi, 17 dpi and 7 dpc |  |
| 10 | C129R | 7 dpi, 13 dpi, 17 dpi and 7 dpc |  |
| 11 | E248R | 7 dpi, 13 dpi, 17 dpi and 7 dpc |  |
| 12 | A137R (p11.5) | 7 dpi and 13 dpi |  |
| 13 | MGF_110_2L |  | 13 dpi, 17 dpi and 7 dpc |
| 14 | I8L | 7 dpi, 13 dpi, and 7 dpc |  |
| 15 | A224L | 7 dpi, 13 dpi and 17 dpi |  |
| 16 | L60L | 7 dpi, 13 dpi, 17 dpi and 7 dpc |  |
| 17 | B438L | 7 dpi, 13 dpi, 17 dpi and 7 dpc |  |
| 18 | MGF_110_12L | 7 dpi and 13 dpi |  |
| 19 | X69R | 7 dpi, 13 dpi, 17 dpi and 7 dpc |  |
| 20 | B407L | 7 dpi, 13 dpi, 17 dpi and 7 dpc |  |
| 21 | MGF_505_3R | 7 dpi and 7 dpc |  |

Table S2. Functional annotations of the ASFV proteins (7-8).

| Protein | Annotation |
| --- | --- |
| A104R | Structural protein/Genome replication, transcription, and translation/Host cell interactions |
| A118R | Transmembrane protein |
| A137R | Structural protein/Host cell interactions |
| A151R | Enzymes or chaperones/Host cell interactions |
| A179L | Host cell interactions |
| A224L | Structural protein/Host cell interactions |
| A238L | Host cell interactions |
| A240L | Genome replication, transcription, and translation |
| A859L | Genome replication, transcription, and translation |
| B117L | Structural protein/Transmembrane protein |
| B119L | Structural protein/Enzymes or chaperones |
| B125R | Uncharacterized protein |
| B169L | Structural protein/Transmembrane protein |
| B175L | Genome replication, transcription, and translation |
| B263R | Genome replication, transcription, and translation |
| B318L | Transmembrane protein/Enzymes or chaperones |
| B354L | Genome replication, transcription, and translation |
| B385R | Genome replication, transcription, and translation |
| B407L | Uncharacterized protein |
| B438L | Structural protein |
| B475L | Transmembrane protein |
| B602L | Enzymes or chaperones |
| B646L | Structural protein |
| B66L | Transmembrane protein |
| B962L | Structural protein/Transmembrane protein/Genome replication, transcription, and translation |
| C122R | Structural protein/Genome replication, transcription, and translation |
| C129R | Structural protein/Host cell interactions |
| C147L | Structural protein/Genome replication, transcription, and translation |
| C257L | Structural protein/Transmembrane protein |
| C315R | Genome replication, transcription, and translation |
| C475L | Structural protein/Genome replication, transcription, and translation |
| C62L | Uncharacterized protein |
| C717R | Structural protein |
| C84L | Uncharacterized protein |
| C962R | Genome replication, transcription, and translation |
| CP123L | Structural protein/Transmembrane protein |
| CP204L | Structural protein/Host cell interactions |
| CP312R | Structural protein |
| CP530R | Structural protein |
| CP80R | Genome replication, transcription, and translation |
| D1133L | Structural protein/Genome replication, transcription, and translation |
| D117L | Structural protein/Transmembrane protein/Host cell interactions |
| D129L | Uncharacterized protein |
| D205R | Structural protein/Genome replication, transcription, and translation |
| D250R | Enzymes or chaperones |
| D339L | Structural protein/Genome replication, transcription, and translation |
| D345L | Genome replication, transcription, and translation |
| DP238L | Uncharacterized protein |
| DP60R | Transmembrane protein |
| DP63R | Uncharacterized protein |
| DP71L | Host cell interactions |
| DP79L | Uncharacterized protein |
| DP96R | Host cell interactions |
| E111R | Uncharacterized protein |
| E120R | Structural protein/Host cell interactions |
| E146L | Structural protein/Transmembrane protein |
| E165R | Structural protein/Genome replication, transcription, and translation |
| E183L | Structural protein/Transmembrane protein/Host cell interactions |
| E184L | Structural protein |
| E199L | Structural protein/Transmembrane protein/Host cell interactions |
| E248R | Structural protein/Transmembrane protein |
| E301R | Genome replication, transcription, and translation/Host cell interactions |
| E423R | Structural protein |
| E66L | Transmembrane protein/Host cell interactions |
| EP1242L | Structural protein/Genome replication, transcription, and translation |
| EP152R | Structural protein/Transmembrane protein |
| EP153R | Structural protein/Transmembrane protein/Host cell interactions |
| EP296R | Structural protein/Genome replication, transcription, and translation |
| EP364R | Genome replication, transcription, and translation/Host cell interactions |
| EP402R | Structural protein/Transmembrane protein/Host cell interactions |
| EP424R | Structural protein/Genome replication, transcription, and translation |
| EP84R | Structural protein/Transmembrane protein |
| F1055L | Genome replication, transcription, and translation |
| F165R | Transmembrane protein |
| F317L | Structural protein/Host cell interactions |
| F334L | Genome replication, transcription, and translation |
| F778R | Genome replication, transcription, and translation |
| G1211R | Genome replication, transcription, and translation |
| G1340L | Structural protein/Genome replication, transcription, and translation |
| H108R | Structural protein/Transmembrane protein |
| H124R | Structural protein |
| H171R | Structural protein |
| H233R | Transmembrane protein |
| H240R | Structural protein |
| H339R | Structural protein |
| H359L | Structural protein/Genome replication, transcription, and translation |
| I10L | Transmembrane protein |
| I177L | Structural protein |
| I196L | Uncharacterized protein |
| I215L | Enzymes or chaperones/Host cell interactions |
| I226R | Host cell interactions |
| I243L | Genome replication, transcription, and translation |
| I267L | Host cell interactions |
| I73R | Structural protein |
| I7L | Transmembrane protein/Host cell interactions |
| I8L | Uncharacterized protein |
| I9R | Uncharacterized protein |
| K145R | Structural protein |
| K196R | Genome replication, transcription, and translation |
| K205R | Host cell interactions |
| K421R | Structural protein |
| K78R | Structural protein/Genome replication, transcription, and translation |
| KP177R | Structural protein/Transmembrane protein |
| L11L | Transmembrane protein |
| L60L | Uncharacterized protein |
| L83L | Host cell interactions |
| M1249L | Structural protein/Host cell interactions |
| M448R | Structural protein/Genome replication, transcription, and translation |
| MGF_100_1R | Uncharacterized protein |
| MGF_110_12L | Transmembrane protein/Host cell interactions |
| MGF_110_13L | Transmembrane protein/Host cell interactions |
| MGF_110_14L | Host cell interactions |
| MGF_110_1L | Transmembrane protein/Host cell interactions |
| MGF_110_2L | Host cell interactions |
| MGF_110_3L | Transmembrane protein/Host cell interactions |
| MGF_110_4L | Structural protein/Transmembrane protein/Host cell interactions |
| MGF_110_5L_6L | Host cell interactions |
| MGF_110_7L | Host cell interactions |
| MGF_110_8L | Host cell interactions |
| MGF_300_1L | Transmembrane protein/Host cell interactions |
| MGF_300_4L | Host cell interactions |
| MGF_360_10L | Transmembrane protein/Host cell interactions |
| MGF_360_11L | Host cell interactions |
| MGF_360_12L | Host cell interactions |
| MGF_360_13L | Host cell interactions |
| MGF_360_14L | Host cell interactions |
| MGF_360_15R | Host cell interactions |
| MGF_360_16R | Host cell interactions |
| MGF_360_18R | Host cell interactions |
| MGF_360_1L | Transmembrane protein/Host cell interactions |
| MGF_360_21R | Host cell interactions |
| MGF_360_2L | Host cell interactions |
| MGF_360_3L | Transmembrane protein/Host cell interactions |
| MGF_360_4L | Host cell interactions |
| MGF_360_6L | Host cell interactions |
| MGF_360_8L | Host cell interactions |
| MGF_360_9L | Host cell interactions |
| MGF_505_10R | Host cell interactions |
| MGF_505_2R | Host cell interactions |
| MGF_505_3R | Host cell interactions |
| MGF_505_5R | Host cell interactions |
| MGF_505_6R | Host cell interactions |
| MGF_505_7R | Host cell interactions |
| MGF_505_9R | Host cell interactions |
| NP419L | Structural protein/Genome replication, transcription, and translation |
| NP868R | Structural protein/Genome replication, transcription, and translation |
| O174L | Structural protein/Genome replication, transcription, and translation |
| O61R | Structural protein/Transmembrane protein |
| P1192R | Genome replication, transcription, and translation |
| p14 | Structural protein |
| p150 | Structural protein |
| p34 | Structural protein |
| p37 | Structural protein |
| Q706L | Structural protein/Genome replication, transcription and translation |
| QP383R | Structural protein/Enzymes or chaperones |
| QP509L | Genome replication, transcription, and translation |
| R298L | Structural protein/Enzymes or chaperones |
| S183L | Uncharacterized protein |
| S273R | Structural protein/Enzymes or chaperones/Host cell interactions |
| X69R | Transmembrane protein |

Table S3. Protein category enrichment analysis of IgG-positive ASFV proteins.

| Protein category |  | Groups B and C | | | | Group B | | | | Group C | | | |
| --- | --- | --- | --- | --- | --- | --- | --- | --- | --- | --- | --- | --- | --- |
|  | Total on array^a^ | Hits | Fold Enrich | *p* value^b^ |  | Hits | Fold Enrich | *p* value |  | Hits | Fold Enrich | *p* value |  |
| Structural proteins | 67 | 30 | 1.56 | 0.000* |  | 25 | 1.81 | 0.000* |  | 27 | 1.7 | 0.000* |  |
| Transmembrane proteins | 38 | 15 | 1.37 | 0.104 |  | 8 | 1.02 | 1.000 |  | 13 | 1.44 | 0.125 |  |
| Genome replication, transcription, and translation | 40 | 7 | 0.61 | 0.074 |  | 6 | 0.73 | 0.373 |  | 6 | 0.63 | 0.197 |  |
| Other enzymes or chaperones | 9 | 2 | 0.77 | 1.000 |  | 2 | 1.08 | 1.000 |  | 1 | 0.47 | 0.687 |  |
| Host cell interactions | 63 | 20 | 1.1 | 0.592 |  | 13 | 1 | 1.000 |  | 18 | 1.2 | 0.260 |  |
| External structural proteins**^c^** | 16 | 12 | 2.61 | 0.000* |  | 9 | 2.73 | 0.001* |  | 11 | 2.89 | 0.000* |  |
| Total | 160 | 46 |  |  |  | 33 |  |  |  | 38 |  |  |  |

^a^The number of annotated proteins included in the microarray for each category. ^b^Asterisks denote significant values. ^c^External structural proteins include proteins located in the outer membrane, capsid, and inner membrane of the virion.

Table S4. Protein category enrichment analysis of IgG-positive ASFV proteins.

| Cluster | Proteins |
| --- | --- |
| Cluster_S1 | A104R, B169L, B646L, CP123L, D117L, D205R, E120R, E184L, KP177R |
| Cluster_S2 | CP204L, CP312R, CP530R, E183L, p150, p34 |
| Cluster_S3 | A224L, B962L, EP153R, MGF_110_4L, O61R |
| Cluster_S4 | B438L, C129L, D1133L, E165R, F317L, G1340L |
| Cluster_S5 | A137R, EP402R, H240R, I73R |

Table S5. Enrichment analysis of ASFV proteins of early and late seroconversion in groups B and C.

| Protein category |  | Group B | | | | | | | | Group C | | | | | | | |
| --- | --- | --- | --- | --- | --- | --- | --- | --- | --- | --- | --- | --- | --- | --- | --- | --- | --- |
|  |  | Early seroconversion^a^ | | |  | Late seroconversion^b^ | | |  | Early seroconversion | | |  | Late seroconversion | | |  |
|  | Total on array | Hits | Fold Enrich | *p* value^c^ |  | Hits | Fold Enrich | *p* value |  | Hits | Fold Enrich | *P*  value |  | Hits | Fold Enrich | *p* value |  |
| Structural proteins | 67 | 27 | 1.5 | 0.002* |  | 3 | 2.39 | 0.072 |  | 14 | 1.34 | 0.129 |  | 16 | 1.82 | 0.000* |  |
| Transmembrane proteins | 38 | 15 | 1.47 | 0.059 |  | 0 | 0 | 1.000 |  | 11 | 1.85 | 0.019* |  | 4 | 0.8 | 0.785 |  |
| Genome replication, transcription and translation | 40 | 7 | 0.65 | 0.151 |  | 0 | 0 | 0.574 |  | 3 | 0.48 | 0.133 |  | 4 | 0.76 | 0.598 |  |
| Other enzymes or chaperones | 9 | 2 | 0.83 | 1.000 |  | 0 | 0 | 1.000 |  | 1 | 0.71 | 1.000 |  | 1 | 0.85 | 1.000 |  |
| Host cell interactions | 63 | 20 | 1.18 | 0.279 |  | 0 | 0 | 0.279 |  | 14 | 1.42 | 0.076 |  | 6 | 0.73 | 0.342 |  |
| Total | 160 | 43 |  |  |  | 3 |  |  |  | 25 |  |  |  | 21 |  |  |  |

^a^Defined to be no later than 7 dpi. ^b^Defined to be later than 7 dpi. ^c^Asterisks denote significant values.
